# Analysis of the co-translational assembly of the fungal fatty acid synthase (FAS)

**DOI:** 10.1038/s41598-020-57418-8

**Published:** 2020-01-21

**Authors:** Manuel Fischer, Mirko Joppe, Barbara Mulinacci, Ronnald Vollrath, Kosta Konstantinidis, Peter Kötter, Luciano Ciccarelli, Janet Vonck, Dieter Oesterhelt, Martin Grininger

**Affiliations:** 1 Institute of Organic Chemistry and Chemical Biology, Buchmann Institute for Molecular Life Sciences, Goethe University Frankfurt, Max-von-Laue-Str. 15, 60438 Frankfurt am Main, Germany; 2 Department of Membrane Biochemistry, Max Planck Institute of Biochemistry, Am Klopferspitz 18, 82152 Martinsried, Germany; 3 Institute of Molecular Genetics and Cellular Microbiology, Goethe University Frankfurt, Max-von-Laue-Str. 9, 60438 Frankfurt am Main, Germany; 4 Department of Structural Biology, Max Planck Institute of Biophysics, Max-von-Laue-Str. 3, 60438 Frankfurt am Main, Germany

**Keywords:** Biocatalysis, Protein folding

## Abstract

The yeast fatty acid synthase (FAS) is a barrel-shaped 2.6 MDa complex. Upon barrel-formation, two multidomain subunits, each more than 200 kDa large, intertwine to form a heterododecameric complex that buries 170,000 Å^2^ of protein surface. In spite of the rich knowledge about yeast FAS in structure and function, its assembly remained elusive until recently, when co-translational interaction of the β-subunit with the nascent α-subunit was found to initiate assembly. Here, we characterize the co-translational assembly of yeast FAS at a molecular level. We show that the co-translationally formed interface is sensitive to subtle perturbations, so that the exchange of two amino acids located in the emerging interface can prevent assembly. On the other hand, assembly can also be initiated via the co-translational interaction of the subunits at other sites, which implies that this process is not strictly site or sequence specific. We further highlight additional steps in the biogenesis of yeast FAS, as the formation of a dimeric subunit that orchestrates complex formation and acts as platform for post-translational phosphopantetheinylation. The presented data supports the understanding of the recently discovered prevalence of eukaryotic complexes for co-translational assembly, and is valuable for further harnessing FAS in the biotechnological production of aliphatic compounds.

## Introduction

**Figure 1:**
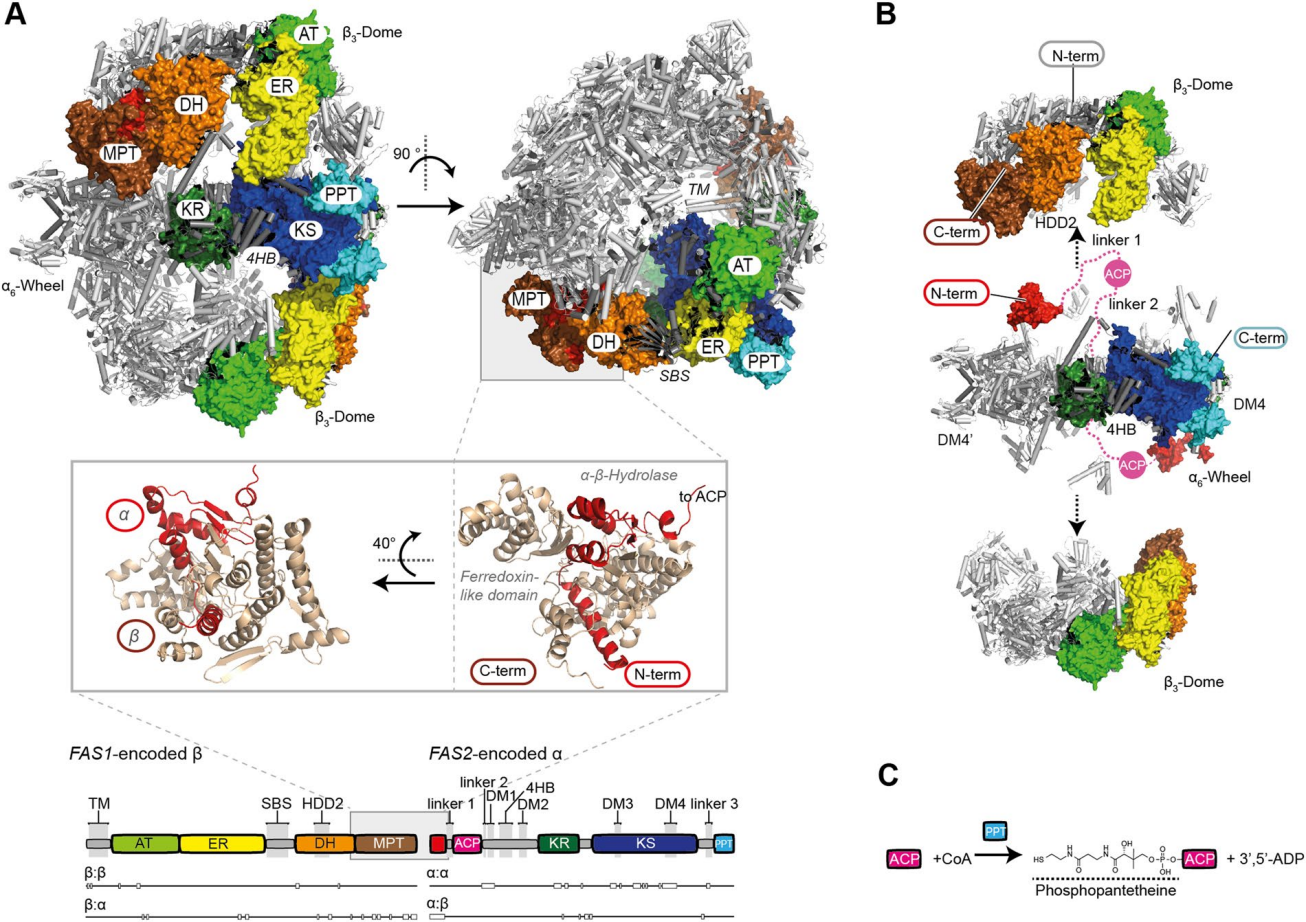
Structure of yeast FAS. (**A**) Structure of *S. cerevisiae* FAS (PDB-code: 3hmj)^17^. Cartoon representation of the X-ray crystallographic structure shown in side (left) and top view (right) with two β-subunits and two α-subunits highlighted by domains in surface representation. ACP is located in the FAS interior, but is not shown in this figure. The MPT fold is comprised of both subunits and shown in cartoon representation (β part in brown and its α part in red). A domain structure is attached indicating interfaces of subunits. Nomenclature: acetyl transferase (AT), enoyl reductase (ER), dehydratase (DH), malonyl-palmitoyl-transferase (MPT), acyl carrier protein (ACP), ketoacyl reductase (KR), ketoacyl synthase (KS) and phosphopantetheine transferase domain (PPT). Insertion elements are highlighted in grey; trimerization module (TM), 6-stranded β-sheet (SBS), hotdog-domain 2 (HDD2), dimerization module 1–4 (DM1-4), 4-helical bundle (4HB)). Please note that DM2 is not visible in this structure. (**B**) Dissection of the yeast FAS barrel into the D3-symmetric α hexamer (α_6_-wheel) and the two C3-symmetric β trimers (β_3_-domes). β_3_-domes have been shifted for clarity (see arrows). View and coloring as in (**A**). ACP domains are shown for two α-subunits, and are modeled by spheres in magenta. ACP linkers are indicated by dashed lines. (**C**) Scheme of the post-translational modification of ACP. For phosphopantetheinylation, ACP and PPT have to physically interact.

Fatty acid synthases (FAS) have been structurally studied during the last years, and a deep understanding about the molecular foundations of *de novo* fatty acid (FA) synthesis has been achieved^1–4^ (Supplementary Fig. 1A,B). The architecture of fungal FAS was elucidated for the proteins from *Saccharomyces cerevisiae* (baker’s yeast)^5–7^ and the thermophilic fungus *Thermomyces lanuginosus*^8^, revealing an elaborate 2.6 MDa large α_6_β_6_ barrel-shaped complex that encapsulates fungal *de novo* FA synthesis in its interior (Fig. 1A). The functional domains are embedded in a scaffolding matrix of multimerization and expansion elements. Acyl carrier protein (ACP) domains, shuttling substrates and intermediates inside the reaction chamber, achieve compartmentalized synthesis^5,9^ (Fig. 1B,C). The concept of metabolic crowding makes fungal FAS a highly efficient machinery, running synthesis at micromolar virtual concentrations of active sites and substrates^10,11^. The outstanding efficacy in fungal FA synthesis is documented by (engineered) oleagenic yeast that can grow to lipid cellular contents of up to 90%^12^. Fungal FAS have also raised interest as biofactories in microbial production of value-added compounds from saturated carbon chains^13–15^.

Notwithstanding a profound knowledge about this protein family, the biogenesis of fungal FAS has not been investigated until recently, when Shiber *et al*. identified yeast FAS as initiating assembly via the co-translational interaction of subunits α (encoded by *FAS2*) and β (*FAS1*)^16^. Co-translational assembly was analyzed with a modified version of a ribosome profiling protocol in which ribosome protected mRNA footprints of nascent chains interacting with “partner proteins” were made visible. A strong enrichment of footprints on the α-subunit starting at amino acid 125 suggested that an N-terminal structural motif of the α-subunit is engaged by the β-subunit for co-translational substructure folding. Footprint data made perfectly sense from a structural perspective, because the C-terminus of β and the N-terminus of α intertwine (about 95 amino acids) to form the MPT domain (see Fig. 1A).

Following our research program to explore the structure and function of multidomain (type I) FAS, and in the light of our specific interest in engineering FAS for custom compound synthesis, we recently started the project of deciphering yeast FAS assembly mechanisms. We were particularly interested in two aspects. Based on the recent study of the co-translational assembly, we aimed at characterizing this interaction in its molecular foundations; i.e., specifying the interface of subunits that foster the interaction and probing the constraints underlying this process. As a second focus, we sought to spotlight yeast FAS assembly in its spatial and temporal coordination with the post-translational modification of the protein. For activating FAS, a phosphopantetheine transferase (PPT), that is located at the perimeter of the FAS barrel, phosphopantetheinylates the ACP (see Fig. 1B,C). The spatial separation of the ACP/PPT functional pair in yeast FAS barrel suggests that phosphopantetheinylation does not occur in the mature, FA synthesis competent α_6_β_6_ state, but in a different structural frame prior to barrel formation^6,17^. As the inconsequent phosphopantetheinylation would be energetically costly, assembly pathways must be particularly dedicated to warranting quantitative phosphopantetheinylation.

This study was greatly aided by engineering the protein on the basis of the available atomic resolution models^5–8,17^. Wildtype and several engineered yeast FAS constructs were analyzed in their ability to complement a FAS-deficient yeast strain and tested for their structural integrity. Full-length and truncated yeast FAS constructs were further recombinantly expressed in *Escherichia coli*. These tools in hand, we were able to address FAS assembly in a “forward-approach”, which means that instead of often-performed dissociation based (“reverse”) approaches, we generated information based on halted assembly states and truncated structures.

Here, we present a multitude of data describing the key processes in the assembly of yeast FAS. This mega-dalton molecule assembles autonomously without the need for additional factors via a single dominant pathway, which is possible by distributing the complexity of the assembly process onto a sequence of domain-domain interactions that are formed one after another. The initial co-translational assembly step relies on the formation of a α-helical bundle-like substructure within the MPT domain, but our data suggests that co-translational assembly can also be initiated at other sites. While the β-subunit is chaperoning α during co-translational substructure formation, the barrel-shaped structure is mainly developed by interactions of domains located on the α-subunit. The dimerization of the KS is a key interaction in this process, providing the platform for the post-translational modification of the ACP before it is encapsulated in the interior of the FAS barrel to serve substrates shuttling.

## Results

### Analysis of the co-translational assembly of yeast FAS

**Figure 2:**
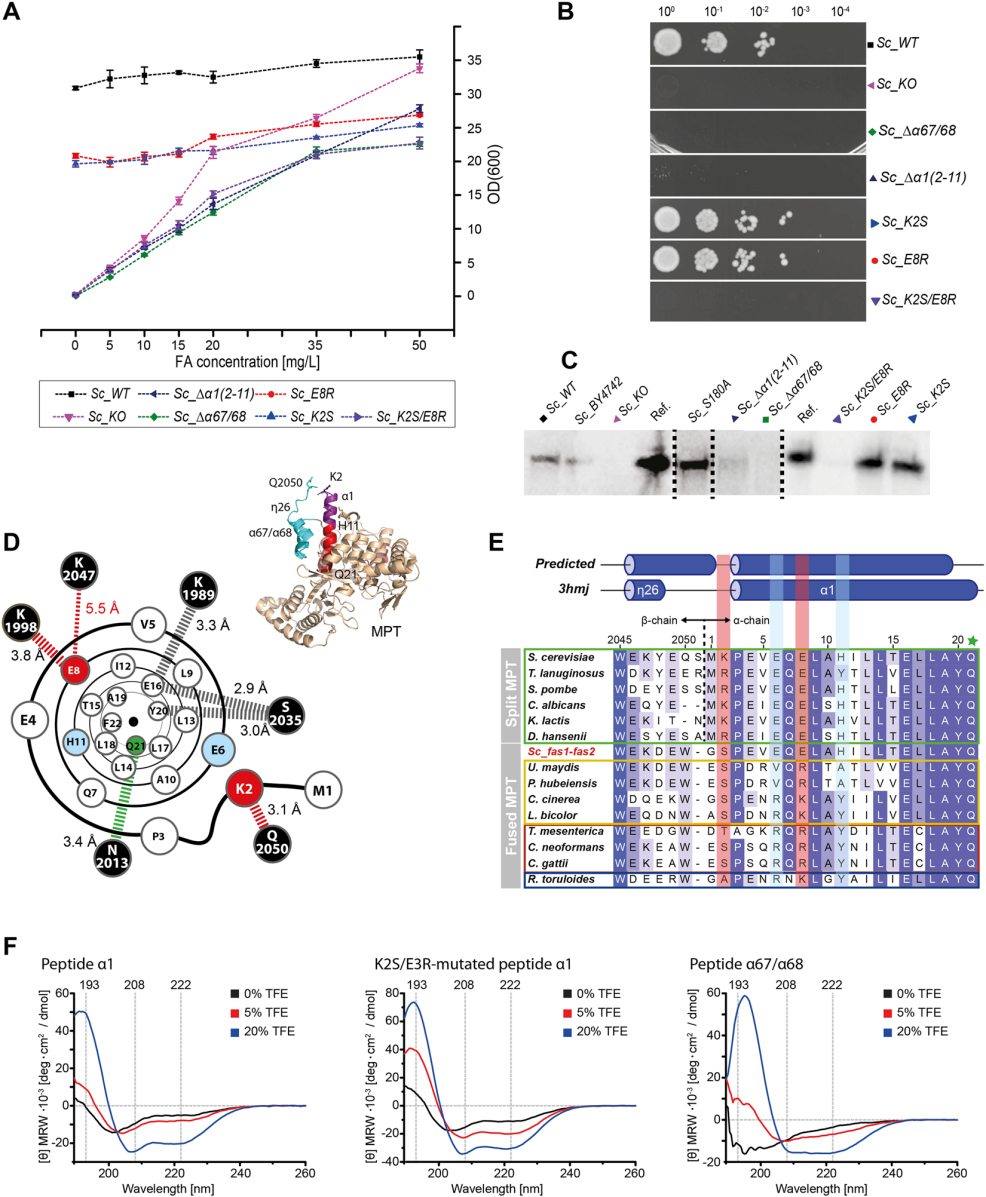
Interaction of α and β during co-translational assembly. For biological replicates and data of additional mutants not shown in this figure see Supplementary Fig. 3A–C and Tables S2. **(A**) Growth behavior of mutated strains in liquid cultures supplemented with external FA. Values were determined  in 5 technical replicates (error bars represent ± 3 σ). Data points are connected by dashed lines for clarity. Please note that the relatively higher ODs for WT and KO originate from deviant starting conditions, as they were precultured in YPD-FA instead of SD-FA medium (see Supplementary Information). (**B**) Ten-fold serial dilutions (starting from OD(600) = 1) of log-phase cultures spotted on YPD agar without external FA supply after incubation for 48 h at 30 °C. (**C**) Native-PAGE-Western-Blot analysis of FAS from mutant strains grown to the log-phase. Bands indicate presence or absence of intact FAS barrels. Purified FAS from *S. cerevisiae* was used as reference (termed Ref). The wildtype strain BY4247 is included as control (termed *Sc_BY4247*). For clarity, the figure has been assembled from different cropped blots as indicated by dashed lines. For the uncropped blots, see Supplementary Fig. 4A–C. (**D**) Cartoon illustrating the α1-helix with key polar interactions to the β (black spheres) represented by dashed bars. Distances as indicated are calculated from the X-ray structure (PDB-code: 3hmj)^17^. Amino acids that were sensitive to mutations in the FAS assembly process are shown as red spheres; insensitive amino acids are shown as blue spheres, and the catalytic Q21 (involved in the catalytic triad of the MPT active site) as green sphere. The MPT domain of yeast FAS is shown for clarity (top left). (**E**) Alignment of sequences covering the α1-helix (*S. cerevisiae* FAS numbering). Sequences include *Ascomycota*-type FAS (green box), single-gene encoded fungal FAS (yellow), *Tremellomycetes*-type FAS (red), *Rhodosporidium*-type FAS (blue) and the engineered *fas1-fas2*-fusion strain *Sc-fas1-fas2*. The alignment was prepared with the program Clustal Omega on the EBI webserver based on the full length FAS sequence and colored according to occurrence^45^. Two-genes encoded FAS were submitted as *FAS1*-*FAS2*-fusions. Predicted *S. cerevisiae* FAS secondary structure from PsiPred^46^ and the secondary structure as observed in the X-ray crystal structure (PDB-code: 3hmj) are attached. Loci that are mutation sensitive in *Ascomycota*-type FAS assembly are highlighted by a red background; two further loci, which we have exchanged in mutational studies are in blue, and the catalytically relevant Q21 is indicated by a green star. Uniprot (or GenBank in case of *Tremella mesenterica*) accession numbers of sequences are: *Candida albicans* (P34731, P43098), *Coprinopsis cinerea* (A8NUB3), *Cryptococcus gattii* (E6R622, E6R621), *Cryptococcus neoformans* (Q5KG98, Q5KG99), *Debaryomyces hansenii* (Q6BWV8, Q6BWN1), *Kluyveromyces lactis* (Q6CWN6, Q6CT25), *Laccaria bicolor* (B0D9Q1), *Pseudozyma hubeiensis* (R9P8H2), *Rhodosporidium toruloides* (M7WSW5, M7XM89), *Saccharomyces cerevisiae* (P07149, P19097), *Schizzosaccharomyces pombe* (Q9UUG0, Q10289), *Thermomyces lanuginosus* (A4VCJ6, A4VCJ7), *Tremella mesenterica* (XP_007006732.1, XP_007006745.1), *Ustilago maydis* (A0A0D1C5S0). (**F**) Analysis of custom-synthesized peptide fragments CD-spectroscopy recorded at different TFE concentrations; peptides α1, K2S-E8R-mutated α1 and in α67/α68.

In order to analyze the co-translational assembly of subunits as an early step in yeast FAS assembly, we generated a set of mutants that modulate the interface of subunits in the co-translationally formed substructure. In the experimental procedure, a FAS-deficient *S. cerevisiae* strain, growing on external FA, was complemented by plasmids encoding the yeast FAS variants (Table S1)^18,19^. Complementation efficiency was read-out by growth rates in FA-limited liquid cultures and by spot dilutions on medium without added FA. We initially tested two FAS constructs in their propensity for co-translational assembly; (i) β deleted in the C-terminal helices α67 and α68 (pRS415_*fas1Δ*α67/α68) combined with wildtype α (pRS413_*FAS2*) yielding strain *Sc_Δα67/α68*, and (ii) wildtype β (pRS415_*FAS1*) combined with α deleted in about half of the N-terminal α1-helix (amino acids K2 to H11; pRS413_*fas2Δα1(2–11)*) giving strain *Sc_Δα1(2–11)*. The latter strain, *Sc_Δα1(2–11)*, is a minimal version of the previously reported assembly deficient FAS mutant that misses the entire N-terminal MPT domain segment of α^16^. The FAS constructs reduce the α/β interface in the MPT (3380 Å^2^) by 520 Å^2^ (2860 Å^2^; *FAS2Δα1(2–11)*) and 460 Å^2^ (2920) Å^2^; *FAS1Δ*α67/α68), respectively. Both of these constructs did not or just very poorly restore *de novo* FA synthesis in the FAS-deficient yeast strain (Fig. 2A,B). We also probed whether absent activity can indeed be attributed to an assembly defect or is rather caused by a putatively compromised catalytic activity of overall intact yeast FAS. For analysis, we blotted cell lysates of the complemented FAS-deficient yeast strains separated by Native-PAGE, and made yeast FAS visible with polyclonal rabbit anti-FAS antibodies^20^. Both strains, *Sc_Δα67/α68* and *Sc_Δα1(2–11)*, did not show FAS bands in Native-PAGE-Western-Blot analysis (Fig. 2C), interpreted as true failure in the assembly of subunits. We expect that non-assembled yeast FAS escapes immunoblotting owing to absent epitopes. Earlier studies have moreover shown that α is rapidly degraded in mutants lacking β^20,21^, so that the cytoplasm of yeast strains with assembly-defective FAS presumably contains only low levels of subunit α. Absence of assembled FAS, as a result of transcriptional down-regulation or RNA instability instead of assembly failure, can be excluded for *Sc_Δα1(2–11)* based on a previous study showing constant expression of a fas2-lacZ fusion-gene lacking the first 39 nucleotides of the FAS2 open reading frame^22^. Please note that details and abbreviations of FAS constructs are outlined in Table 1.

**Table 1:** Yeast FAS strains used in this study (complementation data presented in Fig. 2A–C, Supplementary Fig. 2A–C and Table S2).

Strains	FAS genotype (on pRS, see Table S1)
Sc_KO	∆fas1/∆fas2
Sc_WT	FAS1/FAS2
Sc_K2S	FAS1/fas2_K2S
Sc_E8R	FAS1/fas2_E8R
Sc_K2S/E8R	FAS1/fas2_K2S/E8R
Sc_∆α1(2-11)	FAS1/fas2_∆α1(2-11)
Sc_∆α67/α68	fas1_∆α67/α68/FAS2
Sc_S180A	FAS1/fas2_S180A
Sc_K2S/E6V	FAS1/fas2_K2S/E6V
Sc_K2S/H11 A	FAS1/fas2_K2S/H11A
Sc_E6V/E8R	FAS1/fas2_E6V/E8R
Sc_E6V/H11A	FAS1/fas2_E6V/H11A
Sc_fas1-α1	fas1-α1/FAS2
Sc_fas1-full-MPT	fas1_full-MPT/FAS2
Sc_Tre	fas1Tre/fas2Tre
Sc_Tre_E8R	fas1Tre_E2058R/fas2Tre
Sc_Tre_∆α10-12	fas1Tre/fas2Tre_∆α10-12
Sc_fas2Tre-α9	fas1Tre/fas2Tre-α9
Sc_Rho	fas1Rho/fas2Rho
Sc_Rho_∆(1–53)	fas1Rho/fas2Rho∆(1–53)
Sc_fas1-fas2	fas1-fas2 -fusion
Sc_Asc/Tre	FAS1/fas2∆(428–1887)/fas2Rho

As a next step in the analysis of the subunit interaction, we designed constructs, in which β is elongated for internal competition in the co-translational substructure formation. The elongation of the β-subunit by the 11 amino acids long α1-helix fragment (generating strain *Sc_fas1-α1*) compromised the ability to restore FA *de novo* synthesis. The fusion of the MPT-part encoded on the α-subunit (strain *Sc_fas1-full-MPT*) eventually entirely abolished *de novo* FA synthetic activity indicating successful competitive inhibition of co-translational substructure formation (see Supplementary Fig. 2A–C).

Finally, we mutated the MPT interface of yeast FAS and cloned constructs with point mutations in helix α1 (Fig. 2D**)**. Amino acids K2, E6, E8 and H11 were selected as candidates based on their conservation in *Ascomycota*-type FAS, and mutated to their most frequent exchanges in non-*Ascomycota*-type FAS (Fig. 2E). All single mutated constructs (pRS415_*FAS1*; mutated pRS413_fas2*; K2S, E6V, E8R and H11A) were able to restore *de novo* FA synthesis in the FAS deficient yeast strain. However, double mutated constructs, permutating the above amino acid exchanges, identified K2S-E8R-double mutated FAS as assembly deficient (see Fig. 2A–C). To better understand the impact of mutations, we analyzed custom-synthesized peptide fragments in their secondary structure by CD-spectroscopy in co-solvents^23^. We observed a high propensity of α1-peptide to form a α-helix, which was even more pronounced in the K2S-E8R-mutated peptide (Fig. 2F). The C-terminal α67/α68 fragment of β is less α-helically structured than the α1-peptide. According to this data, the assembly defect of the K2S-E8R mutation, as observed in the complementation assay, may either originate from mutations changing specific interactions during co-translational substructure formation, or from changed α-helical properties of the mutated α1-peptide that interfere in assembly. In any of these two cases, this data shows that the co-translational substructure formation is sensitive to subtle changes in the interface of subunits. Biological replicates of complementation experiments and additional data are shown in Supplementary Fig. 3A–C and Table S2. Original Western Blots are shown in Supplementary Fig. 4A–C.

### The fungal FAS family is topologically heterogeneous on gene level

**Figure 3:**
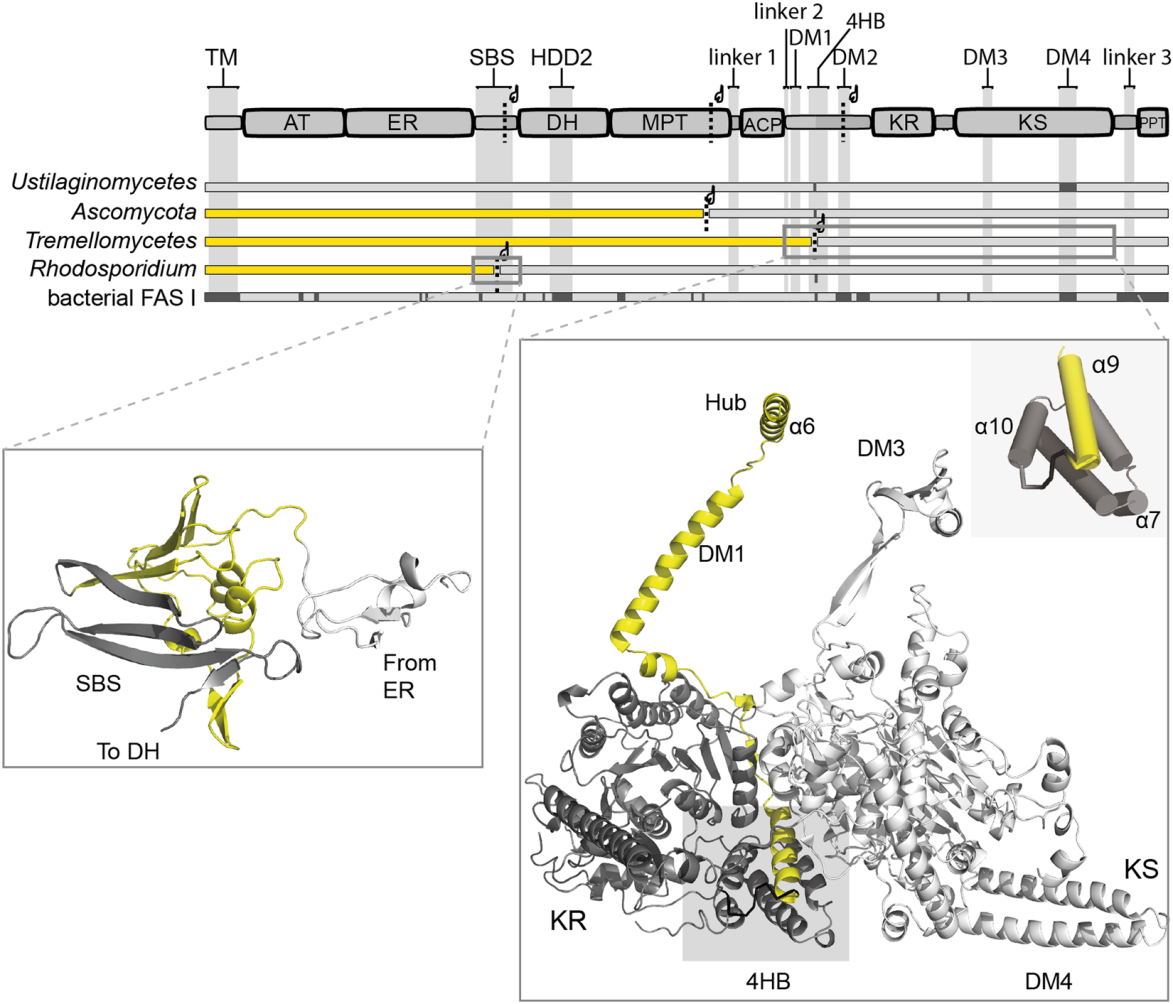
Domain alignment of the fungal FAS family. Topological variants of fungal FAS. Domain architecture is given for single-gene encoded fungal FAS. Abbreviations used as in Fig. 1. Four fungal FAS variants differing in subunit topology and the bacterial type I FAS are given. Missing domains/insertions in the FAS variants are indicated in dark grey. *Ustilaginomycetes*-type FAS carries all domains on a single polypeptide, *Ascomycetes*-type (including *S. cerevisiae* and *C. albicans*), *Tremellomycetes*-type (including *C. neoformans* and *C. gattii*) and *Rhodosporidium*-type FAS are two-gene encoded variants. Substructures that are shown in detail below are highlighted by grey frames in the domain alignment. Yellow and grey coloring indicate *FAS1*-encoded polypeptides (β) and *FAS2*-encoded polypeptides (α), respectively. Secondary structure elements are shown in yeast FAS numbering as introduced by Jenni *et al*.^8^. For an extended version of this figure see Supplementary Fig. 5A–C.

Genome sequence analysis characterizes fungal FAS as a heterogeneous family comprising different gene-topological variants (Fig. 3). As most evident gene-topological variation, fungal FAS are either encoded by single genes or by two genes. Two-gene encoded fungal FAS appear to originate from a single-gene encoded precursor split into two parts at various splitting sites that are generally located within domains^24,25^. In *S. cerevisiae* and *T. lanuginosus* FAS, both representing the *Ascomycota*-type FAS, the C-terminus of β and the N-terminus of α intertwine to form the MPT domain (see Fig. 1A and Supplementary Fig. 5A)^5,8^. In *Tremellomycetes*-type FAS, the termini of polypeptide chains intertwine to form a 4-helical bundle (4HB) at the interface of the KR and the KS domain (see Fig. 3 and Supplementary Fig. 5B). At the *Rhodosporidium toruloides* FAS splitting site, subunits share an antiparallel β-sheet (SBS) domain, but, different to *Ascomycota*- and *Tremellomycetes*-type FAS, termini do not intertwine^26^ (see Fig. 3 and Supplementary Fig. 5C).

The fungal FAS variants were rebuilt in yeast FAS by initially engineering a single-gene encoding fungal FAS with *FAS1* and *FAS2* connected by a sequence that natively links the two genes in *Ustilago maydis* FAS (*Sc_fas1-fas2*) (see Fig. 2E). Taking this construct as a template, we then engineered splitting sites as occurring in *Tremellomycetes*-type (*Sc_Tre*) and *Rhodosporidium*-type FAS (*Sc_Rho*) and probed the constructs in the complementation assay. All three constructs successfully complemented the deficiency in *de novo* FA synthesis of the FAS-deficient yeast, as well as assembled to the barrel-shaped complex. Interestingly, even tripartite yeast FAS, carrying the native splitting site as well as the splitting site of *Tremellomycetes*-type FAS (strain *Sc_Asc/Tre*), successfully assembled to the active and intact protein (Supplementary Fig. 2A–C). We also modulated the interface of subunits in the two-gene encoding variants *Sc_Tre* and *Sc_Rho*. We omitted the N-terminal three α-helices of the *Tremellomycetes*-type mimicking FAS α-subunit and the N-terminal β-sheet of the *Rhodosporidium*-type mimicking FAS α- subunit giving strains *Sc_Tre_∆α10-12* and *Sc_Rho_∆(1–53)*, respectively. Both strains failed to restore *de novo* FA synthesis (see Supplementary Fig. 2A–C). Biological replicates of complementation experiments and additional data are shown in Supplementary Fig. 3A–C and Table S2. Original Western Blots are shown in Supplementary Fig. 4A–C.

While this data shows that yeast FAS assembles to active complexes even when the subunit subdivision occurs elsewhere than at the original site, the experimental set-up cannot disclose whether assembly in FAS with alternative splitting sites is initiated already during translation. However, for *Tremellomycetes*-type FAS, it is plausible to assume co-translational interaction of subunits for the following reasons. First, as found in baker’s yeast (*Ascomycota*-type) FAS, the splitting site is located within a structural motif (4HB). Termini of subunits at this site are highly intertwined, so that this interface can likely not be built from folded subunits (see Fig. 3 and Supplementary Fig. 5B). Second, *Tremellomycetes*-type mimicking FAS is sensitive towards the truncation of the N-terminus of α by two helices (*Sc_Tre_∆α10-12*). This interface contributes only marginally to the overall about 170,000 Å^2^ of protein surface being buried upon barrel formation. Therefore, the sensitivity to the truncated terminus is in line with the co-translational formation of the 4HB motif at the onset of assembly. The situation is unclear for the *Rhodosporidium*-type mimicking FAS. Subunits in *Rhodosporidium*-type FAS are not intertwined (see Supplementary Fig. 5C**)**, and it is tempting to speculate that also post-translational assembly may be possible. In such a scenario, trimers of subunit β (just harboring domains AT and ER, see Fig. 3) build the caps that close the barrel-like core.

### Post-translational modification occurs within a dimeric sub-structure

In a stepwise deconstruction approach, we dissected yeast FAS into domains and multi-domain constructs, which we then analyzed in structural properties and catalytic activity (see Table S1). Since the proteolytic degradation of yeast FAS subunits has been reported as a regulatory step of α/β expression^20^, we produced proteins recombinantly in *E. coli*. We demonstrated the suitability of *E. coli* as an expression host by successfully producing yeast FAS and an *Ustilaginomycetes*-type mimicking *fas1*-*fas2* fusion protein (Supplementary Fig. 6A,B). This agrees with earlier findings on the successful expression of fungal FAS from *R. toruloides*^26^ as well as of the yeast FAS homologous bacterial type I FAS occurring in *Corynebacteria*, *Mycobacteria* and *Nocardia* (CMN-bacterial FAS)^27,28^ (Supplementary Fig. 6C,D). For the deconstruction approach, we focused on the α-subunit. The α-subunit harbors the relevant domains for post-translational modification as well as the domains contributing most of the overall 170,000 Å^2^ buried surface of yeast FAS. The β-subunit was not proteolytically stable as a separate protein in *E. coli*, which impaired *in vitro* assembly experiments.

**Figure 4:**
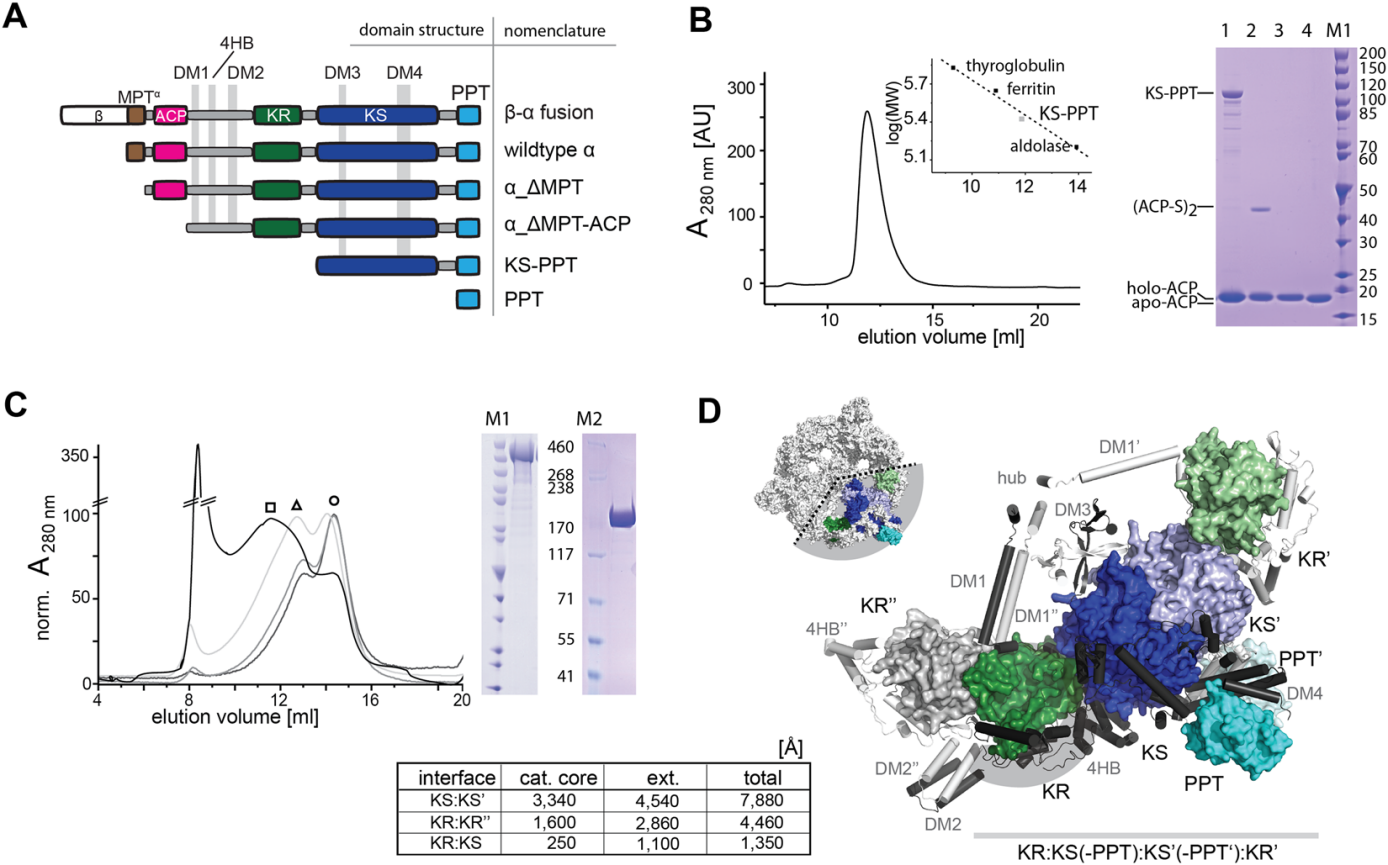
Oligomeric requirements of domains of the α-subunit. (**A**) Overview of constructs expressed in *E. coli* for analysis of yeast FAS assembly. (**B**) Analysis of the KS-PPT di-domain construct. (left panel) SEC on a Superdex 200 Increase 30/100 GL column with calibration curve. The peak corresponds to an apparent molecular weight of 250 kDa, equivalent to a stoichiometry of 2.5 (calculated molecular weight 99.5 kDa), suggesting a dimer with increased apparent weight possibly due to a non-globular shape of the protein. (right) 4–12% Bis/Tris SDS-PAGE gel of a phosphopantetheinylation assay. Holo-ACP tends to dimerize *via* a disulfide formation, giving (ACP-S)_2_, which can be used as read-out for PPT activity. The disulfide bond is cleaved under the reducing conditions of the sample loading buffer. Lane M, marker; 1, reaction solution; 2, ACP purified from the reaction solution and loaded on gel under non-reducing conditions; 3, same as 2 but loaded on gel under reducing conditions; 4, apo-ACP reference. More data to these constructs (the full SDS-PAGE gel) is presented in Supplementary Fig. 7A. (**C**) Analysis of the αΔMPT-ACP construct. (left) SEC on a Superose 6 Increase 30/100 GL column (in TRIS-HCl buffer N100) with protein preparations from purifications under native (black line) and denaturing (grey lines) conditions. Peaks correspond to apparent molecular weights of 400 kDa (○), 600 kDa (∆) and 800 kDa (□). The sharp peak at about 8 ml can be assigned to protein aggregates eluting in the void volume. (right) SDS-PAGE gels of purified αΔMPT-ACP. For marker band M1 see panel B. For the full SDS-PAGE gels see Supplementary Fig. 7C. **(D**) KR:KS(-PPT):KS’(-PPT’):KR’ substructure and analysis of interfaces. Catalytic cores are colored as introduced in Fig. 1A. Insertions are shown in cartoon representation in black. Interfaces are listed as table, and numbers are given for the catalytic cores (cat. core) and the contributions by insertion elements (ext.). For stabilizing the KR:KS interface, a large insertion, including the DM1-4HB connecting linker and 4HB, enwraps the KR (insertion highlighted by grey background). The α_6_-wheel substructure is shown for clarity (top left). Calculation of interfaces and their representation in this figure are based on *S. cerevisiae* FAS data^17^ with modeled DM2^8^.

In order to trace the phosphopantetheinylation competent unit, we probed the role of the C-terminal PPT as a separate domain and as part of larger constructs (Fig. 4A). As has been reported before, the ACP is monomeric, whereas the PPT domain is only active in a multimeric state^17^. As a separate domain, PPT occurs as a trimer^17^, as also described for the bacterial homolog AcpS^29^. SEC analysis of a KS-PPT di-domain construct revealed a dimeric character of the KS-PPT substructure, demonstrating that the large dimeric interface of the KS dimer overrides the PPT trimeric preference (Fig. 4B). Constructs PPT and KS-PPT were phosphopantetheinylation-active (see Fig. 4B and Supplementary Fig. 7A,B), indicating that the PPT domain is active in both its dimeric and trimeric state. While ACP and PPT have to physically interact during phosphopantetheinylation, we were not able to identify stable ACP:PPT complexes by pull-down, co-purification and crosslinking experiments, indicating that the ACP:PPT interaction is transient and unstable (Supplementary Fig. 8A–C).

The N-terminal elongation of the KS-PPT construct by a sequence including DM1, DM2 and KR (construct termed α_ΔMPT-ACP) led to protein aggregation (Fig. 4C). SEC analysis resulted in a sharp peak at an apparent mass of approximately 450 kDa, which corresponds to the molecular weight of a dimeric species, but mainly showed unspecific higher oligomerization/aggregation. SEC elution fractions of α_ΔMPT-ACP still showed PPT-activity, which implies that structured dimeric KS-PPT cores remain intact upon aggregation (see Supplementary Fig. 7B**)**. Higher ratios of dimeric species were received, when the protein was purified under denaturing conditions and refolded by SEC or dialysis under low protein concentrations (see Fig. 4C). Further N-terminal elongation to a α_ΔMPT construct as well as to full-length α did not impede aggregation formation. Intriguingly, in spite of aggregation, the ACP domain of α_ΔMPT was quantitatively phosphopantetheinylated (*in cis*). This was probed by inserting a TEV-proteolytic cleavage site in the linker C-terminal to ACP, allowing ESI-MS analysis of the separate ACP received after TEV-proteolytic digestion of α_ΔMPT aggregates (Supplementary Fig. 9).

Data collected on the truncated α constructs implies that the phosphopantetheinylation active species is dimeric, organized by the KS dimer as the prominent structural unit. It can further be concluded that the sequence ACP-KR-KS-PPT bears the information for forming the phosphopantetheinylation competent complex, but not for forming the D3 symmetric α_6_-wheel structures. For more information on the timing of the post-translational modification, we analyzed the phosphopantetheinylation-deficient S180A yeast FAS in our assembly assay (see Fig. 2C). The mutated construct was unable to restore *de novo* FA synthetic activity in the complementation assay, as expected when abolishing substrate shuttling by ACP. However, the mutated FAS still assembled to the α_6_β_6_ complex, demonstrating that a successful post-translational phosphopantetheinylation is not supervised during assembly. This observation is in agreement with earlier data^30^.

### Impact of scaffolding elements on yeast FAS assembly and stability

**Figure 5:**
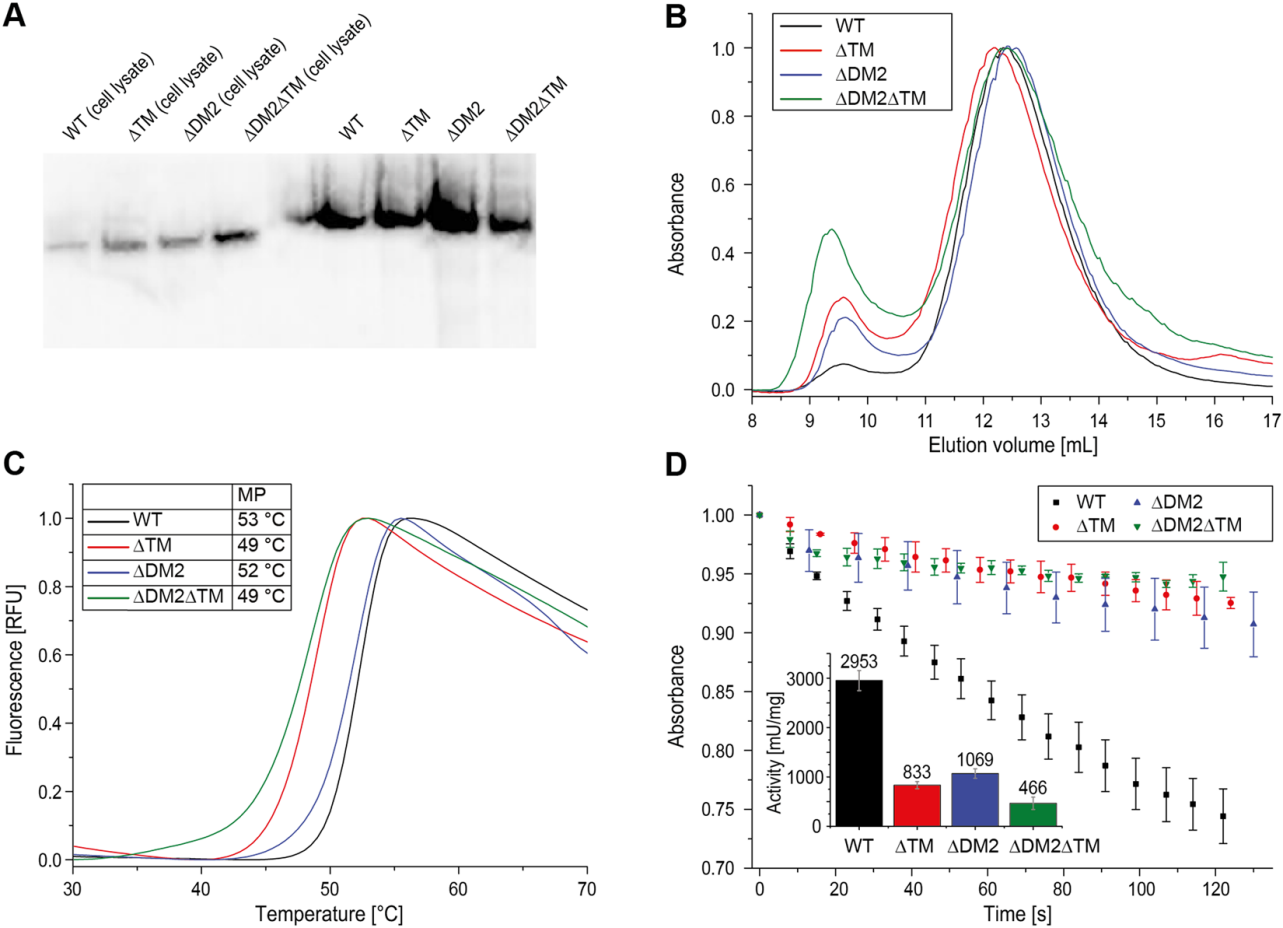
Purification and analysis of yeast FAS with deletion of insertion elements. Abbreviations: WT, *Sc_WT*; ΔDM2, *Sc_ΔDM2*; ΔTM, *Sc_ΔTM*; ΔDM2ΔTM, *Sc_ΔDM2ΔTM* (for more information about strains see Table S1). (**A**) Native PAGE Western Blot of yeast FAS constructs from cell lysates (left) and after purification (right) as indicated. (**B**) FAS constructs as shown in panel A were purified with SEC (Superose 6 Increase 10/300 GL, buffer: 100 mM sodium phosphate pH = 6.5, 200 mM sodium chloride). UV absorption at 280 nm has been normalized. (**C**) Typical melting curves of FAS constructs received in TSA. Fluorescence has been normalized. Melting temperatures (MP) are indicated as average of two technical replicates. The difference in MP between technical replicates was smaller than 0.5 °C. (**D**) Activity assay of FAS constructs shown as time course of NADPH absorption at 334 nm. The calculated specific activities (in mU/mg) depicted as bars. Average and standard deviation (±1 σ) of three technical replicates are shown for each construct.

In order to evaluate the impact of insertion elements on yeast FAS assembly, we further engineered yeast FAS constructs deficient in the trimerization module TM or/and the dimerization module DM2 (see Fig. 1A). The TM closes the barrel at its apical sites and DM2 is placed at the outer barrel perimeter. DM2 increases the KR/KR interface as shown in the crystal structure of *T. lanuginosus* FAS^8^. Native PAGE Western Blot analysis indicates intact assembly of the deletion mutants (Fig. 5A). Further, protein properties were determined *in vitro* on the purified proteins by performing size exclusion chromatography (SEC), a thermal shift assay (TSA) and an enzymatic activity assay (Fig. 5B–D). SEC and TSA data shows compromised stability of the proteins with deleted insertion elements, documented by an increased tendency to aggregation in SEC and a drop in protein melting temperature in TSA. A decrease in overall FA synthesizing activity from initial 2953 ± 205 mU/mg for wildtype FAS to 833 ± 73 mU/mg for the ΔTM deletion (strain *Sc_ΔTM*), 1069 ± 97 mU/mg for the ΔDM2 deletion (*Sc_ΔDM2*) and to 466 ± 125 mU/mg for the double deletion (*Sc_ΔDM2ΔTM*) well correlates with protein stability measures. Data indicates that the insertion modules TM and DM2 are not essential for assembly, but stabilize the FAS barrel and increase the catalytic efficacy. This finding agrees with the distribution of scaffolding elements in the fungal FAS family (see Fig. 3). The trimerization module (TM) and the dimerization modules (DM2 and DM4) are not uniformly distributed within the extended fungal FAS family that includes ancestral variants and the evolutionarily related bacterial type I FAS. These scaffolding elements are, therefore, not essential during the assembly process, but were inserted for stabilizing the elaborate structure after the assembly to the barrel-shaped complex had already been evolved^24,25^.

## Discussion

The co-translational interaction of subunits α and β represents the initial step in the assembly of yeast FAS^16^. The N-terminus of α, likely already developed in its secondary structure as indicated by CD-spectroscopic data, intertwines with the C-terminus of β by getting sandwiched between a structured MPT core fold and a α67/α68 element. The co-translationally formed interaction is sensitive to perturbations as indicated by two experimental set-ups. First, site directed mutagenesis revealed that the perturbation of key interactions at the interface can impede assembly (see Fig. 2A–F). Second, in yeast FAS constructs with subunits terminally extended by the interacting segment, this interaction can be competitively inhibited *in cis* (see Supplementary Fig. 2A–C). This study also shows that the assembly of yeast FAS can also proceed when subunit borders are shifted. Linking subunits of yeast FAS at the co-translationally formed interface and introducing splitting sites at two other sites, within the α-helical bundle structure 4HB of subunit α and within the antiparallel β-sheet SBS of β (see Fig. 3), led to intact protein. Even a tripartite construct (strain *Sc_Asc/Tre*), comprising two splitting sites, remained intact (see Supplementary Fig. 2A–C). This data indicates that the co-translational assembly may not be strictly sequence- or site-specific^31,32^, but may originate from a more general mechanism; e.g. in a setting where protein subunits are brought into proximity during translation^33^.

**Figure 6:**
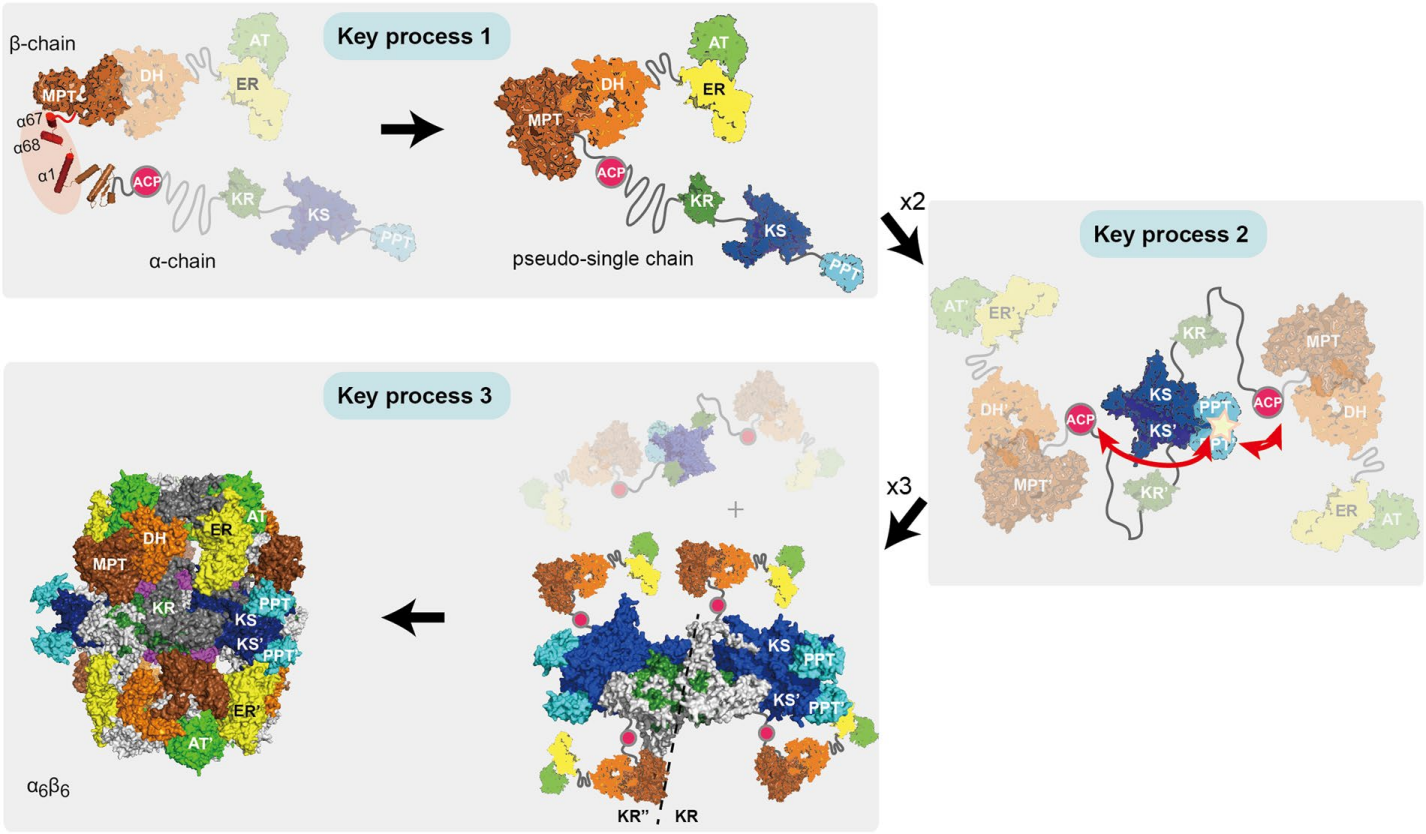
Model for the assembly of yeast FAS in three key processes. The integration of termini for the formation of the MPT proceeds early in assembly. The KS dimerization, that occurs subsequently to process 1, establishes dimeric units that act as platform for the phosphopantetheinylation of ACP by PPT. Finally, abstracted in key process 3, the C2 symmetric dimeric units trimerize to overall D3 symmetric barrel-shaped structures.

The assembly of fungal FAS is strongly constrained by the timing of the phosphopantetheinylation. Since in the mature fungal FAS the ACP is encapsulated in the interior of the protein, while the PPT is located at its perimeter, post-translational modification has to be warranted before barrel enclosure (see Fig. 1B,C)^17^. We have analyzed this aspect by mainly following a deconstruction approach in which we studied the activity of PPT and its interaction with ACP. Our data disclose the dominant structural role of the KS dimer, providing the minimal structural setting for phosphopantetheinylation. While the phosphopantetheinylation can proceed within such a substructure, the α-subunit does not form a stable subcomplex (in *E. coli*), but aggregates unless the β-subunit is present (co-produced in *E. coli*) for steering the assembly towards barrel-formation (see Fig. 4C; for assembled yeast FAS produced in *E. coli* see Supplementary Fig. 6A). Aggregation of subunit α does not impose a problem, because, natively in yeast, α does not exist without being coupled to β. Accordingly, translation of a “β-α-pseudo-single-chain” proceeds until the terminal PPT is released, which is dimerized by the KS for phosphopantetheinylation of ACP. The phosphopantetheinylation status is not read out during assembly, but phosphopantetheinylation proceeds during the course of assembly before barrel enclosure. Most of the insertions elements in yeast FAS contribute to barrel stability, but are not of crucial importance for barrel biogenesis (data have been collected for scaffolding folds DM2 and TM, see Fig. 5A–D). Following this scenario, three processes run in a consecutive order; i.e., the co-translational interaction of α and β forming the MPT domain, the phosphopantetheinylation of ACP structurally orchestrated by the KS dimer, and the barrel enclosure driven by domain-domain interactions but not scaffolding domains (Fig. 6).

The assembly pathway can be correlated to the evolutionary development of fungal FAS, in line with the concept that assembly pathways generally reflect protein evolution^34^. For example, the suggested relevance of the KS dimer for assembly agrees with a recent study that characterized the KS-DM3 dimer as evolutionary ancient^24^, while the scaffolding elements DM2 and TM are not necessary for successful assembly, in line with their appearance at a later stage during fungal FAS evolution when the elaborate barrel-shaped fold had already developed^24,25^. The evolution of two-gene encoded FAS from single-gene encoded FAS is another event that occured at later stage in evolution. The conception of the high evolutionary conservation of assembly pathways in protein families also applies to this detail^35^. Owing to the intial co-translational interaction of subunits, i.e. the co-translational formation of a “β-α-pseudo-single-chain”, all the variants of the heterotopic fungal FAS family (see Figure 3) essentially run through the same assembly pathway as the single-chain evolutionary precursor.

Fungal FAS is the most efficient *de novo* fatty acid (FA) synthesizing protein^10^. This property makes it an attractive target in the endeavor to achieve microbial production of FA and FA derivatives^4,36,37^. The barrel-shaped fold is a suited scaffold that may be repurposed as microbial nano-compartment for harboring also other biosynthetic pathways^38^. Nano-compartments are currently intensively analyzed^11,39^ and harnessed in biotechnological applications^40,41^. The engineering of fungal FAS based nano-compartments require the remodeling of FAS scaffold; i.e., the exchange of the FAS domains by other enzymatic domains, or the insertion of new domain with desired functions. Our data suggests that engineering strategies need to particularly preserve the domains KS and KR (and MPT in *Ascomycota*-type FAS). Domain exchanges to enlarge and modify fungal FAS by new catalytic functions will be difficult to realize, owing to their roles in protein assembly. In contrast, approaches that employ termini or insertion elements as docking or attachment sites may be more promising. The tolerance of yeast FAS towards GFP fusion at the termini of α and β, used for tracing FAS during cell biological studies, can be understood as proof of concept for such an approach^16,42^. Here, our data on the acceptance of alternative splitting sites is goods news for FAS engineering (see Fig. 3 and Supplementary Fig. 2A–C), because the freedom in placing termini allows some spatial control of the positioning of the new domains.

A recent approach has already explored fungal FAS strategies and successfully enlarged the native set of enzymatic domains of yeast FAS by thioesterase domains for the production of short-chain FA and methylketones. The catalytic domains were inserted in the ACP linker sequences as well as at the C-terminus of α^15^. An alternative, recent approach aimed at harnessing the *Ascomycota*-type *Yarrowia lipolytica* FAS as a scaffold for short-chain FA production, by exchanging the MPT fragment at the C-terminus of β with a thioesterase domain. Since interfering in co-translational interaction of subunits, this approach is invasive to the assembly and hinders the formation of intact protein^43^. Such strategies can now be avoided when following the here presented engineering guidelines.

## Methods

Please find a detailed description of the experimental design in **Supplemental Methods**.

### Plasmids and transformation

Yeast plasmids have a pRS backbone with centromeric replication site^18^ and were cloned with homologous recombination in *S. cerevisiae* or with the Infusion HD cloning kit (Clontech, USA) in *E. coli*. All *FAS1* and *FAS2* derived constructs carry the native promoter and terminator sequences^19^. Yeast transformation was done with the LiOAc-method^44^. *E. coli* plasmids have a pET22b backbone (Novagen, USA) and were cloned with the Infusion HD cloning kit (Clontech, USA) (see Table S1).

### Creation of FAS deficient *S. cerevisiae* strain *BY.PK1238_KO*

Strains *Y25032* and *Y21061* were transformed with pMF001 and, after two rounds of sporulation, yielded the haploid *Δfas1; Δfas1* strain. Rejection of the rescue plasmid pMF001 was achieved via selection with 5-fluoroorotic acid.

### Protein purification

Wild type FAS from *S. cerevisiae* as well as ΔTM, ΔDM2 and ΔTM ΔDM2 deletion mutants were isolated as Strep-I-tagged proteins from *S. cerevisiae* with basal expression. Purification of other FAS constructs for *in vitro* studies was achieved from heterologous expressions in *E. coli*.

### Liquid culture growth assay

Cells from single yeast colonies were picked to inoculate 5 mL cultures in appropriate selection medium containing 200 µg/mL geneticin disulfate, free FA (myristic, palmitic and stearic acid, each 50 mg/L) and 1% Tergitol NP-40. After growth at 30 °C and 200 rpm, pre-cultures of same media were inoculated, and grown at 30 °C and 200 rpm to OD(600) 1–14. For 5 mL main cultures in YPD (containing 1% Tergitol NP-40, varying FA concentrations and 200 µg/mL geneticin disulfate), reproducible inocula were obtained by using a standardized inoculum procedure to yield a constant starting OD(600) of 32 × 10^−3^. The cultures were incubated for 24 h at 30 °C and 200 rpm.

### Serial dilution growth assay

Cells were precultured as mentioned above and in 4-fold 1:10 serial dilutions starting from OD(600) 1 transferred onto YPD agar plates without FA. Growth differences were recorded following incubation of the plates for 2–3 days at 30 °C.

### Native PAGE with western blot analysis

*S. cerevisiae* cultures were grown to OD(600) 1 to 2 in appropriate selection medium containing 200 µg/mL geneticin disulfate, free FA (myristic, palmitic and stearic acid, each 50 mg/L) and 1% Tergitol NP-40. Cells were lysed with Zymolyase and lysates were concentrated to total protein concentrations between 1 mg/mL and 5 mg/mL. Native-PAGE (3–12% Bis-Tris gels, Novex, Life Technologies, US) was performed with varying volumes to achieve identical total protein amounts for every sample. As reference, a total amount of 0.1 to 0.2 µg purified *S. cerevisiae* FAS was loaded. After electrophoresis in Blue Native buffer system (Serva Electrophoresis GmbH, Germany) and blotting onto a polyvinylidene difluoride membrane (Immobilon-FL, Merck Millipore, Germany) by electro-transfer, FAS proteins were detected with rabbit anti-FAS antiserum^20^ and horseradish peroxidase conjugated goat anti-rabbit IgG (Pierce, Thermo Fisher Scientific, USA). Luminescence was induced with peroxidase substrate (Carl Roth GmbH, Germany).

### Protein purification and protein biochemical assays

Methods for purification of different FAS constructs and fragments as well as for thermal shift and activity assay are given in the supplementary materials and methods.

### CD-spectroscopy

The peptides α1 (MKPEVEQELAHILLTELLAYQ-NH_2_), α1_K2S/E8R (MSPEVEQRLAHILLTELLAYQ-NH_2_) and α67/α68 (Ac-VTKEYFQDVYDLTGSEPIKEIIDNWEKYEQ) (CASLO ApS, Denmark) were measured at 40 µM in buffer (100 mM NaPi, pH 7.2) with varying volume fractions of 2,2,2-Trifluoroethanol (Alfa Aesar, Johnson Matthey GmbH, Germany) on a Jasco J810 spectrometer (Jasco GmbH, Germany).

## Supplementary information

Supplementary Information.

## Data Availability

The authors will make available all data (underlying the described findings) without restriction.

## References

[1] White SW, Zheng J, Zhang Y-M, Rock CO (2005). The structural biology of type II fatty acid biosynthesis. Annu. Rev. Biochem..

[2] Beld J, Lee DJ, Burkart MD (2015). Fatty acid biosynthesis revisited: structure elucidation and metabolic engineering. Mol. Biosyst..

[3] Herbst DA, Townsend CA, Maier T (2018). The architectures of iterative type I PKS and FAS. Nat. Prod. Rep..

[4] Heil CS, Wehrheim SS, Paithankar KS, Grininger M (2019). Fatty Acid Biosynthesis: Chain-Length Regulation and Control. Chembiochem.

[5] Leibundgut M, Jenni S, Frick C, Ban N (2007). Structural basis for substrate delivery by acyl carrier protein in the yeast fatty acid synthase. Science.

[6] Lomakin IB, Xiong Y, Steitz TA (2007). The crystal structure of yeast fatty acid synthase, a cellular machine with eight active sites working together. Cell.

[7] Johansson P (2008). Inhibition of the fungal fatty acid synthase type I multienzyme complex. Proc. Natl Acad. Sci. USA.

[8] Jenni S (2007). Structure of fungal fatty acid synthase and implications for iterative substrate shuttling. Science.

[9] Gipson P (2010). Direct structural insight into the substrate-shuttling mechanism of yeast fatty acid synthase by electron cryomicroscopy. Proc. Natl Acad. Sci. USA.

[10] Fischer M, Grininger M (2017). Strategies in megasynthase engineering – fatty acid synthases (FAS) as model proteins. Beilstein J. Org. Chem..

[11] Sweetlove LJ, Fernie AR (2018). The role of dynamic enzyme assemblies and substrate channelling in metabolic regulation. Nat. Commun..

[12] Blazeck J (2014). Harnessing Yarrowia lipolytica lipogenesis to create a platform for lipid and biofuel production. Nat. Commun..

[13] Gajewski J (2017). Engineering fatty acid synthases for directed polyketide production. Nat. Chem. Biol..

[14] Gajewski J, Pavlovic R, Fischer M, Boles E, Grininger M (2017). Engineering fungal de novo fatty acid synthesis for short chain fatty acid production. Nat. Commun..

[15] Zhu Z (2017). Expanding the product portfolio of fungal type I fatty acid synthases. Nat. Chem. Biol..

[16] Shiber A (2018). Cotranslational assembly of protein complexes in eukaryotes revealed by ribosome profiling. Nature.

[17] Johansson P (2009). Multimeric options for the auto-activation of the Saccharomyces cerevisiae FAS type I megasynthase. Structure.

[18] Sikorski RS, Hieter P (1989). A system of shuttle vectors and yeast host strains designed for efficient manipulation of DNA in Saccharomyces cerevisiae. Genetics.

[19] Chirala SS (1992). Coordinated regulation and inositol-mediated and fatty acid-mediated repression of fatty acid synthase genes in Saccharomyces cerevisiae. Proc. Natl. Acad. Sci. USA.

[20] Egner R (1993). Tracing intracellular proteolytic pathways. Proteolysis of fatty acid synthase and other cytoplasmic proteins in the yeast Saccharomyces cerevisiae. J. Biol. Chem..

[21] Schüller HJ, Förtsch B, Rautenstrauss B, Wolf DH, Schweizer E (1992). Differential proteolytic sensitivity of yeast fatty acid synthetase subunits alpha and beta contributing to a balanced ratio of both fatty acid synthetase components. Eur. J. Biochem..

[22] Wenz P, Schwank S, Hoja U, Schueller H-J (2001). A downstream regulatory element located within the coding sequence mediates autoregulated expression of the yeast fatty acid synthase gene FAS2 by the FAS1 gene product. Nucleic Acids Res..

[23] Buck M (1998). Trifluoroethanol and colleagues: cosolvents come of age. Recent studies with peptides and proteins. Q. Rev. Biophys..

[24] Bukhari HST, Jakob RP, Maier T (2014). Evolutionary Origins of the Multienzyme Architecture of Giant Fungal Fatty Acid Synthase. Structure.

[25] Grininger M (2014). Perspectives on the evolution, assembly and conformational dynamics of fatty acid synthase type I (FAS I) systems. Curr. Opin. Struct. Biol..

[26] Fischer M (2015). Cryo-EM structure of fatty acid synthase (FAS) from Rhodosporidium toruloides provides insights into the evolutionary development of fungal FAS. Protein Sci..

[27] Ciccarelli L (2013). Structure and conformational variability of the Mycobacterium tuberculosis fatty acid synthase multienzyme complex. Structure.

[28] Enderle M, McCarthy A, Paithankar KS, Grininger M (2015). Crystallization and X-ray diffraction studies of a complete bacterial fatty-acid synthase type I. Acta Crystallogr. F. Struct. Biol. Commun..

[29] Lambalot RH (1996). A new enzyme superfamily - the phosphopantetheinyl transferases. Chem. Biol..

[30] Fichtlscherer F, Wellein C, Mittag M, Schweizer E (2000). A novel function of yeast fatty acid synthase. Subunit alpha is capable of self-pantetheinylation. Eur. J. Biochem..

[31] Panasenko OO (2019). Co-translational assembly of proteasome subunits in NOT1-containing assemblysomes. Nat. Struct. Mol. Biol..

[32] Schwarz A, Beck M (2019). The Benefits of Cotranslational Assembly: A Structural Perspective. Trends Cell Biol..

[33] Mayr C (2018). Protein complexes assemble as they are being made. Nature.

[34] Levy ED, Boeri Erba E, Robinson CV, Teichmann SA (2008). Assembly reflects evolution of protein complexes. Nature.

[35] Marsh JA (2013). Protein complexes are under evolutionary selection to assemble via ordered pathways. Cell.

[36] d’Espaux L, Mendez-Perez D, Li R, Keasling JD (2015). Synthetic biology for microbial production of lipid-based biofuels. Curr. Opin. Chem. Biol..

[37] Hu Y, Zhu Z, Nielsen J, Siewers V (2019). Engineering Saccharomyces cerevisiae cells for production of fatty acid-derived biofuels and chemicals. Open. Biol..

[38] Maier T (2017). Fatty acid synthases: Re-engineering biofactories. Nat. Chem. Biol..

[39] Castellana M (2014). Enzyme clustering accelerates processing of intermediates through metabolic channeling. Nat. Biotechnol..

[40] Wheeldon I (2016). Substrate channelling as an approach to cascade reactions. Nat. Chem..

[41] Zhang Y, Hess H (2017). Toward Rational Design of High-efficiency Enzyme Cascades. ACS Catal..

[42] Shpilka T (2015). Fatty acid synthase is preferentially degraded by autophagy upon nitrogen starvation in yeast. Proc. Natl Acad. Sci. USA.

[43] Xu P, Qiao K, Ahn WS, Stephanopoulos G (2016). Engineering Yarrowia lipolytica as a platform for synthesis of drop-in transportation fuels and oleochemicals. Proc. Natl Acad. Sci. USA.

[44] Gietz RD, Schiestl RH (2007). High-efficiency yeast transformation using the LiAc/SS carrier DNA/PEG method. Nat. Protoc..

[45] Sievers F (2011). Fast, scalable generation of high-quality protein multiple sequence alignments using Clustal Omega. Mol. Sys Biol..

[46] Buchan DWA, Minneci F, Nugent TCO, Bryson K, Jones DT (2013). Scalable web services for the PSIPRED Protein Analysis Workbench. Nucleic Acids Res..

