# Supplementary material for: Analysis of the co-translational assembly of the fungal fatty acid synthase (FAS)
Source: Sci Rep. 2020 Jan 21;10:895. doi: 10.1038/s41598-020-57418-8 (PMC6972935; doi:10.1038/s41598-020-57418-8)
Supplement: Supplementary file 1 — Supplementary Information. [file 41598_2020_57418_MOESM1_ESM.pdf]

SUPPLEMENTARY INFORMATION (SI) APPENDIX

Analysis of the co-translational assembly of the fungal fatty acid synthase (FAS)

Authors: Manuel Fischer[a], Mirko Joppe[a], Barbara Mulinacci[b], Ronnald Vollrath[b], Kosta Konstantinidis[b], Peter Kötter[c], Luciano Ciccarelli[d], Janet Vonck[d], Dieter Oesterhelt[b] and Martin Grininger[a,b]*

Author affiliations: (a) Institute of Organic Chemistry and Chemical Biology, Buchmann Institute for Molecular Life Sciences, Cluster of Excellence for Macromolecular Complexes, Goethe University Frankfurt, Max-von-Laue-Str. 15, 60438 Frankfurt am Main, Germany, (b) Department of Membrane Biochemistry, Max Planck Institute of Biochemistry, Am Klopferspitz 18, 82152 Martinsried, Germany, (c) Institute of Molecular Genetics and Cellular Microbiology, Goethe University Frankfurt, Max-von-Laue-Str. 9, 60438 Frankfurt am Main, Germany.
(d) Department of Structural Biology, Max Planck Institute of Biophysics, Max-von-Laue-Str. 3, 60438 Frankfurt am Main, Germany.

*Corresponding author: grininger@chemie.uni-frankfurt.de

Abbreviations: FA, fatty acids; FAS, fatty acid synthase; PPT, phosphopantetheine transferase; ACP, acyl carrier protein; KS, ketoacyl synthase; KR, ketoacyl reductase; PKS, polyketide synthase

SI METHODS

Chemicals, Enzymes and media

Chemicals were obtained in *p.a.* quality from Alfa Aesar (Johnson Matthey GmbH, Germany) or Sigma Aldrich (Merck KGaA, Germany), unless otherwise indicated. Primers and synthetic DNA fragments were synthesized by Sigma-Aldrich (Merck KGaA, Germany). Enzymes for PCR and plasmid digests were obtained from New England Biolabs (USA). LB media for *E. coli* cultivation were obtained from Carl Roth GmbH & Co. KG (Germany). YPD was prepared with 1% yeast extract, 2% peptone (Becton, Dickinson and Company, USA) and 2% dextrose (Carl Roth GmbH & Co. KG, Germany). For the preparation of YPD-agar plates, 1.5% Agar (Bacto Agar, Becton, Dickinson and Company, USA) was added. SCD media were prepared with *Yeast Nitrogen Base Without Amino Acids*, appropriate *Yeast Synthetic Drop-out Medium Supplements* (Sigma Aldrich, Merck KGaA, Germany) and 2% dextrose (Carl Roth GmbH & Co. KG, Germany). For the liquid culture growth assay, free fatty acids in concentrations as indicated were added to SCD or YPD medium, respectively, from a warmed, ten-fold, aqueous stock solution (myristic, palmitic and stearic acid, each 500 mg/L and 10% Tergitol NP-40).

S. cerevisiae strains

BY4742	*MATα; ura3Δ0; leu2Δ0; his3Δ1; lys2Δ0*
Y25032	*BY4743; MATa/MATα; ura3Δ0/ura3Δ0; leu2Δ0/leu2Δ0; his3Δ1/his3Δ1; met15Δ0/MET15; LYS2/lys2Δ0; YKL182w/YKL182w::kanMX4*
Y21061	*BY4743; MATa/MATα; ura3Δ0/ura3Δ0; leu2Δ0/leu2Δ0; his3Δ1/his3Δ1; met15Δ0/MET15; LYS2/lys2Δ0; YPL231w/YPL231w::kanMX4*
BY.PK1238_KO	*MATα; ura3Δ0; leu2Δ0; his3Δ1; MET15; lys2Δ0; YKL182w/YKL182w::kanMX4; YPL231w/YPL231w::kanMX4*

Creation of FAS deficient *S. cerevisiae* strain *BY.PK1238_KO*

The hetero-diploid, single mutated yeast strains *Y25032* and *Y21061* (both obtained from Euroscarf, http:// http://www.euroscarf.de) were transformed with the *FAS1* and *FAS2* complementing rescue plasmid pMF001, and their geneticin resistant segregants from tetrad dissection were crossed. The resulting hetero-diploid, double mutated strain was sporulated to obtain the haploid *Δfas1; Δfas1* strain. Deletions were verified by tetrad analysis and diagnostic PCR. Rejection of the rescue plasmid pMF001 was achieved via selection on 5-fluoroorotic acid containing (1 g/L) SCD -trp medium supplemented with free fatty acids (myristic, palmitic and stearic acid, each 50 mg/L, 1% Tween20) to yield *BY.PK1238_KO*.

Plasmid constructions

Yeast plasmids: The open reading frames for *FAS1* and *FAS2* were amplified from chromosomal DNA (strain *BY4742*) with their native promoter (995 bp upstream for *FAS1* and 480 bp upstream for FAS2 according to literature) and terminator sequences (295 bp downstream for *FAS1* and 258 bp for *FAS2*) [1]. The rescue plasmid pMF01 was assembled by homologous recombination in *BY4742* from the three fragments *FAS1, FAS2* and pRS416 (Euroscarf, www.euroscarf.de). All other plasmids were cloned and amplified in *E. coli* using the Infusion HD cloning kit (Clontech, USA) yielding plasmids as listed in **Table S1**. For the introduction of point mutations in the α1-comprising part of *FAS2* as well as for the creation of the ACP knockout mutation S180A, the plasmid pMF013 was linearized by PCR with primers excluding a 60-70 nt long section covering the mutation site. Subsequently, plasmids were closed again by inserting synthetic DNA with mutations via Infusion HD cloning kit (Clontech, USA). (see **Table S1**). For construction of plasmids encoding the ΔTM and ΔDM2 deletion constructs, plasmids pMF012 and pMF013 were linearized with primers under removal of codons for amino acid positions 2-137 on *FAS1* (ΔTM) and 541-598 on *FAS2* (ΔDM2), respectively, and closed again using the Infusion HD cloning kit (Clontech, USA).

E. coli plasmids: *S. cerevisiae* PPT as a SUMO-tagged fusion protein, PPT-H(6) and ACP-H(6) were cloned as previously reported [2]. Cloning of other constructs was performed by assembling gel-purified inserts with linearized plasmids via the Infusion cloning kit (Clontech, USA). For domain borders of constructs see **Table S1**: (i) For co-expression plasmid pFAS1_FAS2, *FAS1* and *FAS2* were inserted in pETDuet-1 (Novagen) multiple cloning sites (MCS) 1 and 2, respectively. *FAS2* was equipped with a strep-II tag encoding sequence [3] at the 5'end and a His(6)-tag encoding sequence at the 3'end, yielding an N-terminally strep-II and C-terminally His(6)-tagged β-subunit. pFAS12, encoding a β-subunit-α-subunit fusion construct FAS_β-α (see bridging sequence in Fig. 2E), was created by deleting the MCS1-MCS2 interspacing T7-promotor-2 carrying sequence of pFAS1_FAS2. The β/α fusion construct was equipped with a N-terminal strep-II tag. (ii) MPT truncated FAS2 was encoded by pFAS2_ΔMPT (in pET22b(+) (Novagen) backbone) as N-terminally strep-I [3] and C-terminally H(8)-tagged construct. A TEV cleavage site was introduced at the C-terminus of ACP by site-directed mutagenesis for obtaining pFAS2_ΔMPT_tev (for sequence details see Fig. S8-A).

(iii) pFAS2_ΔMPT-ACP (in pET22b(+) backbone) was designed as N-terminally strep-II and C-terminally H(6)-tagged construct. (iv) pFAS2_KS-PPT (in pGEX-6p-3 (GE Healthhcare) backbone) was constructed as providing a GST-tagged KS-PPT didomain protein. (v) *S. cerevisiae* FAS ACP, as a Sumo-tagged protein, was encoded by pSumo-ACP. The backbone plasmid pET28M-Sumo1 was kindly provided by the European Molecular Biology Laboratory (EMBL) Heidelberg. The ACP mutant S180A was derived from the plasmid pSumo-ACP following a site-directed mutagenesis protocol.

Yeast transformation

For yeast transformations, approximately 1 µg of each plasmid DNA were co-transformed following a modified lithium acetate protocol [4]. A 5 mL over night culture grown in YPD containing 200 µg/mL geneticin disulfate, free FA (myristic, palmitic and stearic acid, each 50 mg/L) and 1% Tween20 at 30 °C and 200 rpm was used to inoculate a 50 mL main culture in the same medium to OD(600) 0.2. After incubating at 30 °C and 200 rpm to OD(600) 0.8, 5 mL of this culture were harvested by centrifugation (3000 *g*, 5 min, 4 °C). During all further steps, cells were kept on ice, unless otherwise indicated. Cells were washed by resuspension in 1 mL water and centrifuged again. After resuspension in lithium acetate solution (0.1 M), cells were incubated for 5 min at 24 °C and centrifuged (5000 *g*, 15 s, 4 °C) before the transformation mix was added (240 µL PEG 1500 solution (50%), 76 µL water, 36 µL lithium acetate solution (1.0 M), 5.0 µL single stranded DNA solution from salmon testis (10 mg/mL), 2.0 µL of each plasmid DNA solution). The cell suspension was mixed and incubated for 30 min at 30 °C followed by 20 min at 42 °C. After pelleting by centrifugation (4000 *g*, 15 s, 4 °C), cells were washed with 1 mL water, pelleted again (4000 *g*, 15 s, 4 °C) and resuspended in 100 µL water. For selection of yeast transformants, the cell suspension was spread on appropriate SCD agar plates containing 200 µg/mL geneticin disulfate, free FA (myristic, palmitic and stearic acid, each 50 µg/mL) and 1% Tween20.

Liquid culture growth assay

Cells from single yeast colonies were picked to inoculate 5 mL cultures in YPD medium (Sc_KO, SC_WT) or respective SCD medium (all other transformants) each containing 200 µg/mL geneticin disulfate, free FA (myristic, palmitic and stearic acid, each 50 mg/L) and 1% Tergitol NP-40. After growth at 30 °C and 200 rpm, pre-cultures of same media were inoculated, and grown at 30 °C and 200 rpm to OD(600) 1-14. For 5 mL main cultures in YPD (containing 1% Tergitol NP-40, varying FA concentrations and 200 µg/mL geneticin disulfate), reproducible inocula were obtained by using a standardized inoculum procedure; i.e. differences in OD(600) were compensated by adjusting inocula volumes, in order to always yield a theoretical starting OD(600) of 32×10^{-3}. The cultures were incubated for 24 h at 30 °C under thorough shaking with 200 rpm. OD(600) values were measured of 20- or 50-fold dilutions in water in 10 mm polystyrene cuvettes (Sarstedt, Germany) with a NanoDrop 2000c spectrophotometer (Thermo Fisher Scientific, USA).

Serial dilution growth assay

Pre-cultures of yeast transformants were prepared according to the liquid culture growth assay (see above). Cells were pelleted (3000 *g*, 5 min, 4 °C), washed with water and resuspended in water to OD(600) 1 followed by 4-fold 1:10 serial dilutions in water. The diluted cultures were transferred with a sterile metal stamp onto YPD agar plates. Growth differences were recorded following incubation of the plates for 2-3 days at 30 °C.

Purification of *S. cerevisiae* FAS and TM/DM deletion mutants from *S. cerevisiae* expression

Strain *BY.PK1238_KO* was transformed with the respective plasmids (see **Table S1**), and cells from a single colony were precultured in 50 mL YPD medium containing 200 µg/mL geneticin disulfate for 2.5 days at 30 °C and 200 rpm to stationary phase. 2 L YPD medium were inoculated from the preculture to a theoretical OD(600) 0.5, and incubated for 10 h at 30 °C and 120 rpm until OD(600) 10. Cells were harvested by centrifugation (4000 *g*, 10 min, 4 °C), washed with 80 mL water and disrupted by beating glass beads in buffer (100 mM NaP_i, pH 7.4; 100 mM NaCl, DNAse-I, 1 tablet/25 mL protease inhibitor cocktail (cOmplete, Roche Diagnostics GmbH, Germany) for 10 x 15 s at 5 m/s with 45 s chilling on ice in between. After sedimentation of glass beads and intact cells (3200 *g*, 10 min, 4 °C), as well as cell debris and insoluble proteins (20000 *g*, 30 min, 4 °C), the supernatant was filtered through glass wool followed by a 22 µm polyethylene styrene filter (Millex, Merck Millipore, Germany). Isolation of the strep-I-tagged FAS was achieved by affinity chromatography with Strep-Tactin resin (IBA GmbH, Germany). Size exclusion chromatography with a Superose 6 increase 10/300 GL column (GE Healthcare, US) in the same buffer was used for further purification and quality check.

<u>Purification of FAS constructs from recombinant expressions</u>

The Sumo-tagged fusion proteins (H(6)Sumo-ACP and H(6)Sumo-PPT) as well as ACP-H(6) were expressed and purified as previously reported [2]. PPT-H(8) was purified similar as described for ACP-H(6), and used because of its better solubility during recombinant expressions in *E. coli* as compared to the previously described PPT-(H6) construct [2].

<u>FAS^{rc} and FAS_β-α:</u> To heterologously co-express α and β subunit in *E. col* (protein termed FAS^{rc}), the plasmid pFAS1_FAS2 was transformed into ArcticExpress Competent Cells (Stratagene, US). Single colonies were used to inoculate 35 mL of LB medium containing 100 µg/mL ampicillin, and incubated at 37°C for 16 h. 20 mL were transferred to 2 L of TB medium supplemented with 100 µg/mL ampicillin, and grown at 30°C to OD(600) 0.9. The culture was incubated at 12°C, and induced with IPTG (0.25 mM final concentration). After incubation at 12°C for additional 16 h, cells were harvested, and lysed in lysis buffer (50 mM Tris-HCl pH 7.36, 10 mM EDTA, 5mM β-ME, 525 (w/v) sucrose, 1 mM PMSF) containing protease inhibitors (cOmplete, Roche Diagnostics GmbH, Germany), DNase (Roche Diagnostics GmbH, Germany), lysozyme, 0.5% (v/v) NP-40 and 5 mM $MgCl_2$. The mixture was incubated at 4 C for 1 h under gentle rotation. The lysate was centrifuged for 1 h at 4°C at 47,000 *g*, and the supernatant was incubated with Ni-NTA Superflow (QIAGEN) for 1 h at 4°C. During purification with gravity flow columns, the protein was washed with 100 mM phosphate buffer pH 6.5 containing 40 mM imidazole and eluted with 100 mM phosphate buffer pH 6.5 containing 500 mM imidazole. Fractions were pooled, concentrated and further purified on Strep-Tactin (washing buffer: 100 mM Tris, pH 8.0, 150 mM NaCl, 1 mM EDTA, elution buffer: 100 mM Tris, pH 8.0, 150 mM NaCl, 1 mM EDT, 2.5 mM desthiobiotin). Ultracentrifugation was used as final purification step [5].

α_ΔMPT-ACP: Plasmid pFAS2_ΔMPT-ACP was transformed into BL21 Gold (DE3) cells (Novagen, US). Single colonies were used to inoculate 35 mL of LB medium containing 100 µg/mL ampicillin, and incubated at 37°C for 16 h. 30 mL were transferred to 3 L of TB medium supplemented with 100 µg/mL ampicillin, and grown at 37°C to OD(600) 0.9. The culture was incubated at 20°C, and induced with IPTG (0.5 mM final concentration). After incubation at 20°C for additional 16 h, cells were harvested, resuspended in buffer N100 (20 mM Tris-HCl pH 7.5, 100 mM NaCl, and 1 mM $MgCl_2$) containing 20 mM imidazole, protease inhibitors (cOmplete, Roche Diagnostics GmbH, Germany), and DNase (Roche Diagnostics GmbH, Germany), and broken by French Press. The lysate was centrifuged for 1 h at 4°C at 47,000 g, and the supernatant was incubated with Ni-NTA Superflow (Qiagen, Germany) for 1 h at 4°C. During purification with gravity flow columns, the protein was washed with 20 mM and eluted with 500 mM imidazole. Fractions containing the target protein were pooled, concentrated and further purified on Strep-Tactin resin (IBA GmbH, Germany; washing buffer: 100 mM Tris, pH 8.0, 150 mM NaCl, 1 mM EDTA, elution buffer: 100 mM Tris, pH 8.0, 150 mM NaCl, 1 mM EDT, 2.5 mM desthiobiotin). Size exclusion chromatography with a Superose 6 10/300 column (GE Healthcare) with buffer N100 was performed as final purification step. α_ΔMPT-ACP was moreover produced in Rosetta2 cells (Life Technologies, US), with the same protocol described above, and obtained in inclusion bodies (IBs). The IBs were resuspended and homogenized in washing buffer (500 mM NaCl, 100 mM phosphate buffer pH 7.5, 20 mM EDTA) containing 1% TritonX-100 and lysozyme. The suspension was centrifuged for 10 min at 4°C at 47,000 g. This treatment was repeating twice and performed another three times with washing buffer only (without TritonX-100 and lysozyme). For purifying α_ΔMPT-ACP under denaturing conditions, the IBs were solubilized in 6 M guanidinium-HCl, 100 mM phosphate buffer pH 7.5, 100 mM NaCl, 20 mM imidazole. Ni-chelating chromatography was performed by washing twice with 20 mM imidazole buffer and finally eluting with 500 mM imidazole. The fractions were pooled and the protein refolded on Superose 6 10/300 column (GE Healthcare, US), or via dialysis in both cases using buffer N100.

GST-KS-PPT: Plasmid pFAS2_KS-PPT was transformed into BL21 Gold (DE3) cells (Novagen, US). Single colonies were used to inoculate 35 mL of LB medium containing 100 µg/mL ampicillin, and incubated at 37°C for 16 h. 10 mL were transferred to 1 L of TB medium in 5 l flask supplemented with 100 µg/mL ampicillin, and grown at 37°C to OD(600) 0.9. The culture was incubated at 15°C, and induced with IPTG (0.5 mM final concentration). After incubation at 15°C for additional 16 h, cells were harvested, resuspended in PBS pH 7.4 containing protease inhibitors (cOmplete, Roche Diagnostics GmbH, Germany), and DNase (Roche Diagnostics GmbH, Germany), and broken by French Press. The lysate was centrifuged for 1 h at 4°C at 47,000 g, and the supernatant was incubated over night with glutathione sepharose 4 Fast Flow (GE Healthcare, US) at 4°C. During purification with gravity flow columns, the resin was washed with PBS pH 7.4 and equilibrated with Prescission Protease Buffer (50 mM Tris-HCl pH 7.0, 150 mM NaCl, 1 mM EDTA, 1 mM DTT)

and incubated with Prescission Protease (GE Healthcare, US; 90 µL per mL of resin) over night at 4°C to remove the GST tag. The protein was then eluted with Prescission Protease Buffer, and size exclusion chromatography with a Superdex 200 10/300 column (GE Healthcare, US) using 20 mM Tris-HCl (pH 7.5), 150 mM NaCl and 1.5 mM $MgCl_2$ buffer was used as final purification step.

α_ΔMPT_tev: This construct was used to provide quantitative data on the efficacy of the phosphopantetheinylation reaction. α_ΔMPT_tev has inserted a TEV cleavage site within the flexible linker region C-terminal to the ACP domain. Treatment of this protein with TEV protease allows ESI-MS analysis of an N-terminal fragment of the α-subunit, which carries the ACP domain. Plasmid pFAS2_ΔMPT_tev was transformed into BL21 Gold (DE3) cells (Agilent, US). Single colonies were used to inoculate 35 mL of LB medium containing 100 µg/mL ampicillin, and incubated at 37°C for 16 h. 30 mL were transferred to 3 L of TB medium supplemented with 100 µg/mL ampicillin, and grown at 37°C to OD(600) 0.9. The culture was cooled on ice to 20°C, and induced with IPTG (0.25 mM final concentration). After incubation at 20°C for additional 16 h, cells were harvested. Cells, resuspended in buffer A (100 mM Tris-HCl pH 8, 100 mM NaCl, 0.1 mM EDTA) containing 10 mM imidazol, protease inhibitors (cOmplete, Roche Diagnostics GmbH, Germany), and DNase (Roche Diagnostics GmbH, Germany)), were broken by French press. The lysate was centrifuged for 1 h at 4°C at 47,000 g. The lysate was incubated 1 h at room temperature with Ni-NTA Superflow (Qiagen, Germany), which was equilibrated with buffer B (20 mM Tris-HCl pH 7.4, 8 M urea) containing 20 mM imidazole. During purification with gravity flow columns, the protein was washed at 20 mM and eluted at 500 mM imidazole. The elution fractions were pooled and dialyzed for 16 h at 4°C against buffer A. The dialyzed solution was incubated with TEV protease for 4 h at 25 °C under gentle shaking. Isolation of the strep-I tagged ACP was achieved by affinity chromatography with Strep-Tactin resin (IBA GmbH, Germany; washing buffer: 100 mM Tris, pH 8.0, 150 mM NaCl, 1 mM EDTA, elution buffer: 100 mM Tris, pH 8.0, 150 mM NaCl, 1 mM EDTA, 2.5 mM desthiobiotin). Denaturation should strip putatively PPT-bound CoA, which could allow posterior phosphopantetheinylation of ACP. Due to the C-terminal purification step, the ACP fragment for analysis exclusively results from full-length α_ΔMPT_tev only (for purification and TEV cleavage, see above).

Thermal Shift Assay (TSA):

All steps were done on ice. For the TSA, 21 μL of buffer (100 mM sodium phosphate buffer pH = 6.5, 200 mM sodium chloride) was added in a white Multiplate® PCR Plate 96 Well™ (Biorad, US) and mixed with 2 μL protein solution (0.9 mg/mL) and 2 μL 62.5 X SYPRO® Orange protein gel stain (Life Technologies, US). The plate was closed with iCycler iQ® Optical Sealing Tape (Biorad, US) and measured with a LFX96 Touch Real Time PCR Detection system (Biorad, US) in the range of 5 °C to 95 °C in steps of 0.5 °C/min (λ_{ex}: = 450-490 nm, λ_{em}: = 560-580 nm).

FAS activity assay:

For the activity assay, 200 mM NaH2PO4/Na2HPO4 (pH = 7.3), 1.75 mM 1,4 Dithiothreitol (Carl Roth, Germany), 0.03 mg/mL BSA (Serva, Germany), 0.7 μg cerFAS, 60 μmol malonyl CoA (Sigma Aldrich, Germany), 50 μmol acetyl CoA (Sigma Aldrich, Germany) and 30 μmol NADPH (Sigma Aldrich, Germany) were mixed in a Costar® 96 Well Cell Culture Plates (Corning®, US) on a 120 μL scale. The background measurement was performed without malonyl CoA. The NADPH consumption was determined by tracking the absorption at 334 nm with a CLARIOstar® Monochromator Microplate Reader (BMG Labtech, Germany) for 130 seconds. The slope was determined with linear regression and was subtracted from the slope of the background measurement. Prior to this, the absorption of NADPH was related to its concentration with calibration, which was used to determine the NADPH consumption. The activity is shown as U/mg where 1U was defined as the incorporation of 1 μmol malonyl-CoA per minute, which is equal to 2 μmol NADPH.

Native PAGE with Western Blot analysis

Enzymatic lysis of *S. cerevisiae* cells: A 5 mL over night culture grown in SCD medium (containing 200 µg/mL geneticin disulfate, free FA (myristic, palmitic and stearic acid, each 50 mg/L) and 1% Tween20) at 30 °C and 200 rpm was used to inoculate a 50 mL main culture in the same medium to OD(600) 0.2. This main culture was incubated at 30 °C and 200 rpm to OD(600) 1 to 2, before cells were harvested by centrifugation (3200 *g*, 8 min, 4 °C). Cells were washed by resuspension in 40 mL ice cold water, pelleted again (3200 *g*, 8 min, 4 °C) and resuspended in „x" mL digestion medium (20 mM Tris-HCL, pH 7.6; 1.1 M sorbitol; 1mM DTT and 57 units/mL zymolyase (AMS Biotechnology, UK)) with „x" meaning 5*OD(600). The mixture was incubated at 30 °C for 2 h with brief, gentle shaking every 30 min. After centrifugation (3200 *g*, 5 min, 4 °C), the pellet was resuspended in 2.5 mL homogenization medium (50 mM Tris-HCl, pH 7.6; 1.1 M glycerol; 5 mM EDTA; 1 mM DTT and 1% of a protease inhibitor cocktail tablet (cOmplete, Roche Diagnostics GmbH, Germany)), and incubated on ice for 2 h. Cell debris were pelleted by centrifugation (3200 *g*, 15 min, 4 °C) and the supernatant applied to Native PAGE.

Western blot analysis: Cell lysates were prepared as described above and concentrated with cellulose membrane centrifugal filters with a 100 kDa cut-off (Amicon Ultra, Merck KGaA, Germany) giving total protein concentrations between 1 mg/mL and 5 mg/mL as determined by light absorbance at 280 nm. Samples were mixed in 1:1 ratio with Blue Native sample buffer (2x) (Serva Electrophoresis GmbH, Germany) and loaded on Native-PAGE 3-12% Bis-Tris gels (Novex, Life Technologies, US) with varying volumes to achieve identical total protein amounts for every sample. As FAS reference, a total amount of 0.1 to 0.2 µg purified *S. cerevisiae* FAS in 2.0 to 4.0 µL of final Blue Native sample was loaded. After electrophoresis (150 V, 2 h, ice cooling) with Blue Native buffer system (Serva Electrophoresis GmbH, Germany), the gel was blotted onto a polyvinylidene difluoride

membrane (Immobilon-FL, Merck Millipore, Germany) by electro-transfer (25 V, 1 h, ice cooling) under buffered conditions (0.5 M bicine; 0.5 M Bis-Tris, pH 7.2; 20 mM EDTA; 10% methanol). The membrane was blocked with I-Block (Thermo Fisher Scientific, USA), and FAS proteins were detected with 1:5000 dilutions of rabbit anti-FAS antiserum [6] followed by incubation with 200 ng/mL horseradish peroxidase conjugated goat anti-rabbit IgG (Pierce, Thermo Fisher Scientific, USA). Luminescence was recorded for 12 to 20 sec (Fusion-SL, Peqlab Biotechnologie GmbH, Germany) after incubation for 5 min at 25 °C with peroxidase substrate (Carl Roth GmbH, Germany).

CD-spectroscopy

The peptides α1 (MKPEVEQELAHILLTELLAYQ-NH_2), α1_K2S/E8R (MSPEVEQRLAHILLTELLAYQ-NH_2) and α67/68 (Ac-VTKEYFQDVYDLTGSEPIKEIIDNWEKYEQ) (CASLO ApS, Denmark) were dissolved in buffer (100 mM NaPi, pH 7.2) with concentrations of 40 µM and varying volume fractions of 2,2,2-Trifluoroethanol (Alfa Aesar, Johnson Matthey GmbH, Germany), and filtered through a 0.22 µm mixed-cellulose-esters filter (Millex, Merck KGaA, Germany). CD-spectra were recorded on a Jasco J810 spectrometer (Jasco GmbH, Germany) in 1 mm quartz cuvettes at 22 °C under nitrogen atmosphere in ten averaged iterations. Background signal for measurements was collected on pure buffers, and used for correction of data.

PPT activity assay

PPT activity assays were performed as previously described [2].

ACP/PPT interaction studies

By size-exclusion-chromatography (SEC): SEC was performed on a SMART chromatography system (Pharmacia; Sweden) equipped with a Superdex 200 PC 3.2/30 column (GE Healthcare, US). ACP and PPT were used in equimolar concentrations of about 0.05 mM each. CoA and 3',5'-ADP were supplied in excess (see Fig. S5-C). Generally, samples were incubated for 10 min at 30 °C under shaking. In case CoA or 3',5'-ADP were used, PPT was first preincubated with compounds before adding ACP. The column was loaded with 50 µ volumes.

By cross-linking: For cross-linking, glutaraldehyde was used as bifunctional reagent. The cross-linking reaction was carried out in 20 mM sodium phosphate buffer, 150 mM NaCl, 5 mM $MgCl_2$. Reactions were performed with equimolar concentrations of ACP and PPT (about 0.05 mM each) and a molar excess of CoA and 3',5'-ADP, respectively (see Fig. S5-A). Total reaction volumes of 50 µL were used. Generally, samples were pre-incubated for 10 min at 30 °C under shaking, before glutaraldehyde was added to a final concentration of 0.05%. After incubating for 10 minutes at 30 °C under shaking, the crosslinking reaction was quenched with a highly concentrated solution of Tris-HCl pH 7.5 buffer. Samples were analyzed by SDS-PAGE.

By Ni-NTA pull-down: In this study, His-tagged ACP as well as His-tagged PPT was used for binding to Ni-NTA beads (Qiagen, Germany). Interaction studies were performed with equimolar concentrations of untagged ACP and PPT (0.05 mM each), and CoA and 3',5'-ADP were supplied in excess (see Fig. S5-B). In order to prevent unspecific binding to the Ni-NTA beads, a buffer was used containing a low concentration of imidazole (20 mM imidazole, 20 mM Tris-HCl pH 7.5, 150 mM NaCl, 5 mM MgCl). The protein mixture (total volume 500 µL) was incubated for 25 minutes at 4 °C (15 minutes preincubation of PPT with CoA and 3',5'-ADP, respectively) and then mixed with 50 µL equilibrated Ni-NTA beads. After one hour of incubation at 4 °C, the mixture was washed three times, and finally eluted with 500 mM imidazole. Samples were analyzed by SDS-PAGE.

SI FIGURES

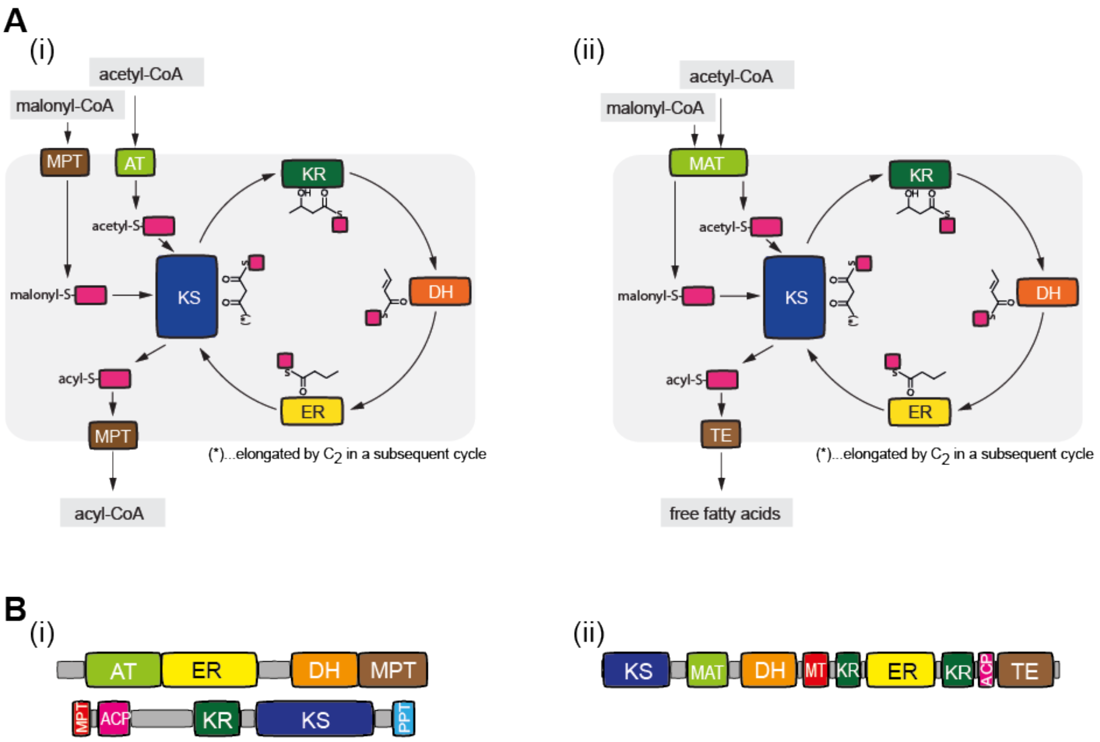

Figure S1. Fatty acid cycle and FAS type I domain architectures. A Fatty acid production as occurring in FAS multienzyme complexes in fungi (**i**) and mammals (**ii**). The grey background highlights compartmentalized synthesis. Substrate CoA-esters are turned into ACP-esters by the transferase domains MPT (malonyl-palmitoyl-transferase) and AT (acetyl transferases), as well as MAT (malonyl-acetyl transferases). ACP (boxes in magenta) then shuttles substrates and intermediates to the catalytic domains. Key steps of the catalytic cycle are as following: The central ketoacyl synthase (KS) domain condenses ac(et)yl with malonyl to form β-ketobutyryl. The condensation product is further reduced to β-hydroxyacyl by a ketoacyl reductase (KR) domain, dehydrated by a dehydratase (DH) domain to produce enoyl, and again reduced by an enoyl reductase (ER) domain. ACP bound acyl chains are cleaved off from the ACP by MPT or TE (thioesterase) yielding different product types (ester or free acid). In fungal FAS, MPT has a dual function and loads malonyl and unloads palmitoyl. **B** Domain structure of fungal FAS (on the example of *Saccharomyces cerevisiae* FAS) (**i**), as well as of mammalian FAS (**ii**). For domain nomenclature, see (A); additional domains are methyltransferase (MT) and phosphopantetheine transferase (PPT). Note, that in yeast FAS, the MPT is shared between the β- (colored in brown) and the α-subunit (red).

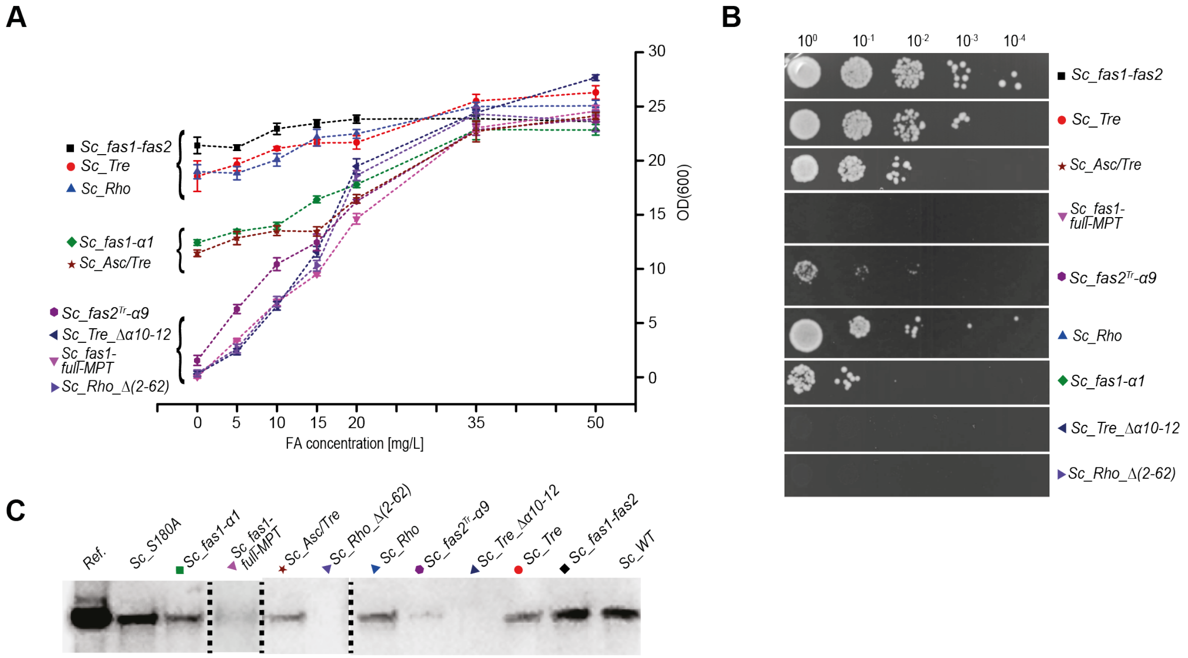

Figure S2. Interaction of subunits during co-translational assembly. Details and abbreviations of complementation constructs are outlined in **Table S2**. Here, we note that the *Tremellomycetes*-type mimicking FAS construct did not include elongations of termini in the splitting site (see **Figure S5B**). **A** Growth behavior of mutated strains in liquid cultures supplemented with external FA. Experimentally determined values (each in 5 technical replicates; error bars represent ±3 σ) are connected by dashed lines for clarity. For biological replicates, see **Figure S3B** and **Table S2**. Strains cluster in different complementation behavior: fully complementing, compromised complementing and non-complementing. **B** Ten-fold serial dilutions of log-phase cultures were spotted on YPD agar without external FA supply and incubated for 48 h at 30 °C. C Native-PAGE-Western-Blot analysis of FAS from mutant strains grown to the log-phase. Bands indicate presence or absence of intact FAS barrels. The figure is assembled from different blots as indicated by dashed lines. The complete blots are shown in **Figure S5A-C**.

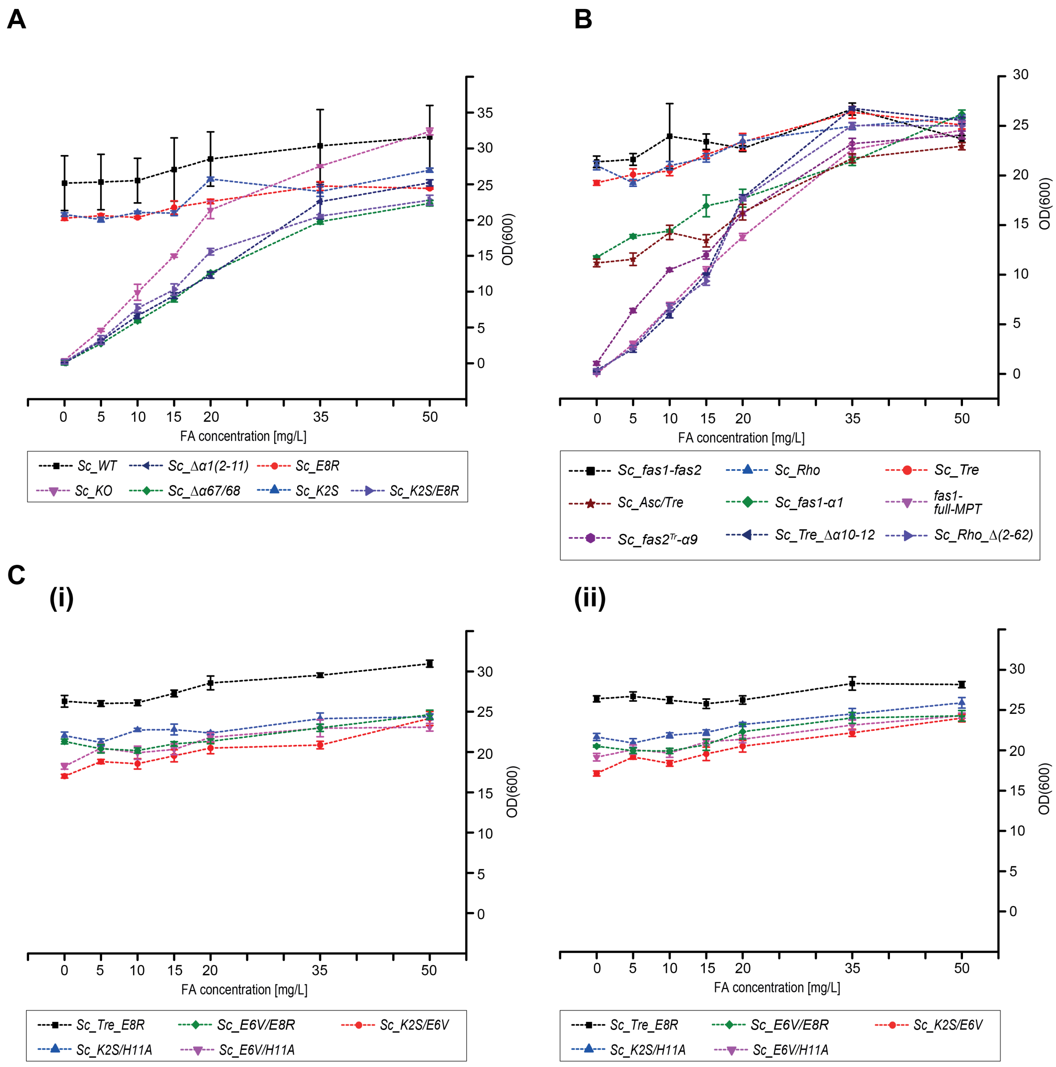

Figure S3. Growth behavior of mutated strains in liquid cultures supplemented with external FA (biological replication and additional data). Experimentally determined values (each in 5 technical replicates) are connected by dashed lines for clarity. Error bars represent ±3 σ. **A** Biological replicates of strains shown in **Figure 2A**. **B** Biological replicates of strains shown in **Figure S2A**. Strains cluster in complementation behavior in fully complementing, compromised complementing and non-complementing. **C** Growth behavior of further mutants in two biological replicates (**i**) and (**ii**), which are discussed in the main text. Please note that the general relatively higher OD(600) for *ScTre_E8R* is resulting from preculturing the strain in YPD- instead of SD-medium as done for all other strains.

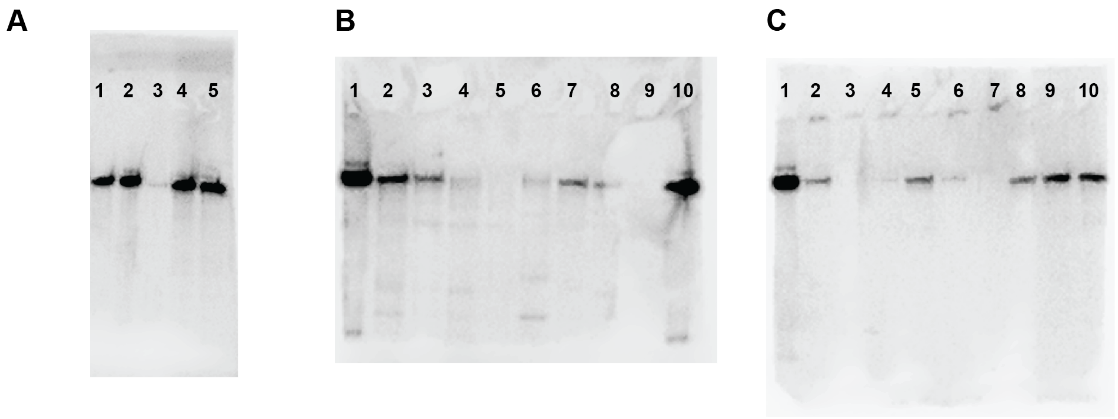

Figure S4. Western Blot of Native-PAGE gels. A lanes 1, *Sc_fas1-fas2*; 2, purified *S. cerevisiae* FAS; 3, *Sc_K2S/E8R*; 4, *Sc_E8R*; 5, *Sc_K2S*. **B** lanes 1, purified *S. cerevisiae* FAS; 2, *Sc_S180A*; 3, *Sc_fas1-α1*; 4, *Sc_Δα1(2-11)*; 5, *Sc_Δα67/68*; 6, *Sc_fas1-full-MPT*; 7, *Sc_WT*; 8, wild type laboratory strain *BY4742*; 9, *Sc_KO*; 10, purified *S. cerevisiae* FAS. **C** lanes 1, purified *S. cerevisiae* FAS; 2, Sc_Asc/Tre; 3, *Sc_Rho_Δ(1-53)*; 4, not from this study; 5, *Sc_Rho*; 6, *Sc_fas2Tr-α9*; 7, *Sc_Tre_Δα10-12*; 8, *Sc_Tre*, 9, *Sc_fas1-fas2*; 10, *Sc_WT*.

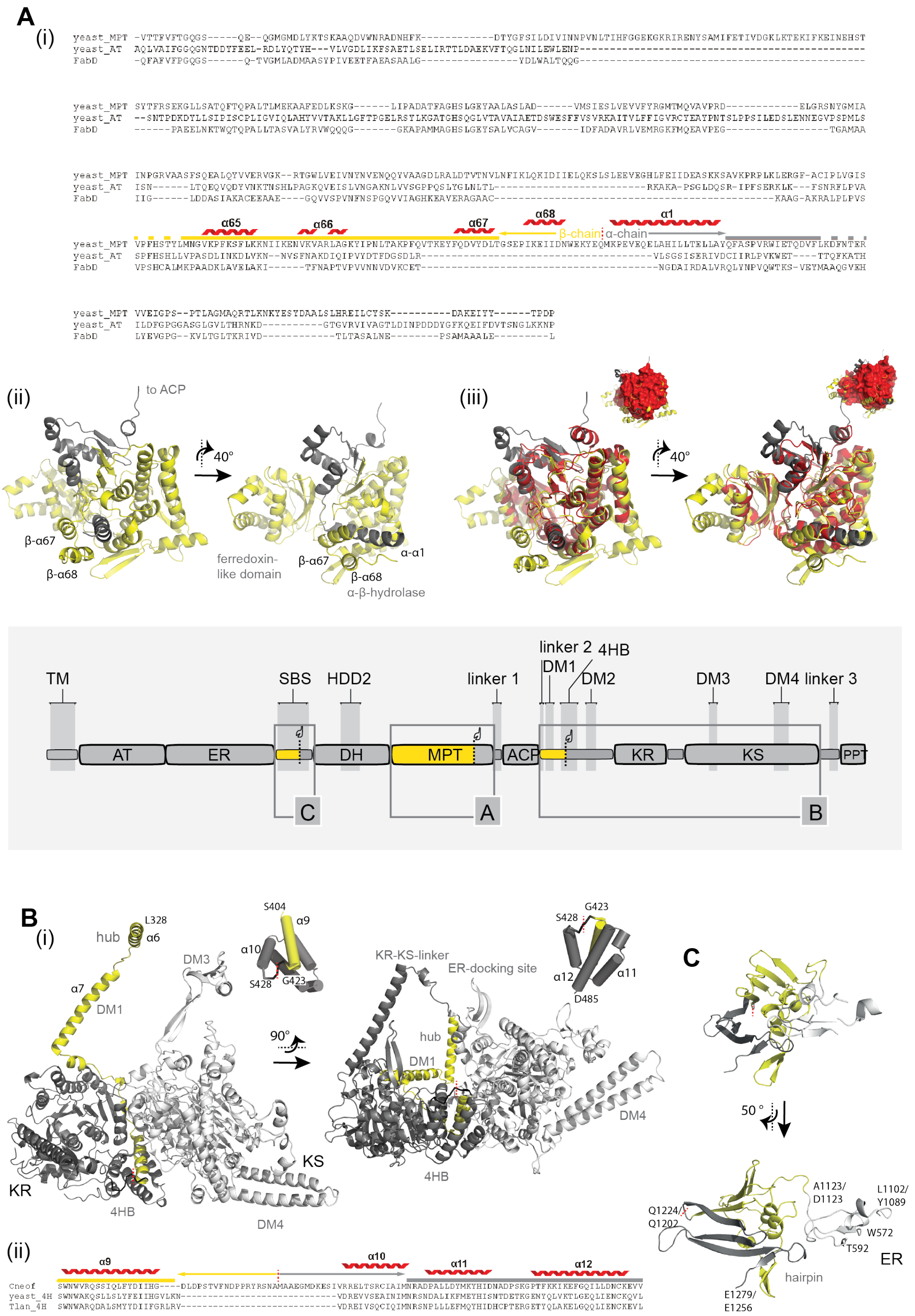

Figure S5. Interaction of subunits in two-genes encoded fungal FAS. As an overview, the domain architecture of fungal FAS is shown in the center of the figure (grey background). For highlighting the composition of domains in fungal FAS variants by two polypeptide chains, the *FAS1* encoded parts are shown in yellow and the *FAS2* encoded parts in grey. The red dashed lines indicate splitting sites. **A** *Ascomycota*-type FAS with the splitting site in the MPT

domain. Sequence alignment of the *S. cerevisiae* MPT (termed yeast MPT) domain with its AT counterpart (yeast AT) and *E. coli* FabD. Secondary structure elements are shown for the interface region. (bottom left) Cartoon representation of the X-ray structural model of the MPT domain [7] with the β- and α-subunit part colored in yellow and grey, respectively. (bottom right) *E. coli* FabD X-ray structural model superimposed [8]. Superposition reveals scaffolding domains as insertions in a FabD-like core fold. Insets show FabD in surface representation. **B** *Tremellomycetes*-type FAS with the splitting site in the 4HB at the interface of domains KR and KS (KS domain shown in light grey). Cartoon representation of the KR-KS substructure of the α-subunit of *S. cerevisiae* FAS carrying the 4HB element [7]. Coloring in yellow and grey represent polypeptide borders as occurring in *C. neoformans* FAS. The α9/α10 connecting loop is shown in black for indicating the splitting site (see also red dashed line). In *Tremellomycetes*-type FAS, the interface is likely enlarged by terminal elongations. The sequence alignment of the *C. neoformans* and *S. cerevisiae* FAS at the 4HB element is attached. Highlighted secondary structure motifs are in *S. cerevisiae* FAS numbering. **C** The SBS element carries the splitting site in *Rhodosporidium*-type FAS (*T. lanuginosus* model; pdb codes 2uva, 2uvb) [9]. The interface is comprised by the formation of a curved β-sheet assembled by both subunits that interacts with α-helices of the Fas1-encoded subunit at the concave face. A hairpin loop of is further interacting with a globular fold of the ER domain (colored in light grey). Residue numbering according to *T. lanuginosus /S. cerevisiae* FAS, which were correlated by sequence alignment [10]. Secondary structure elements in panels A to C are numbered according to Jenni *et al.* [9].

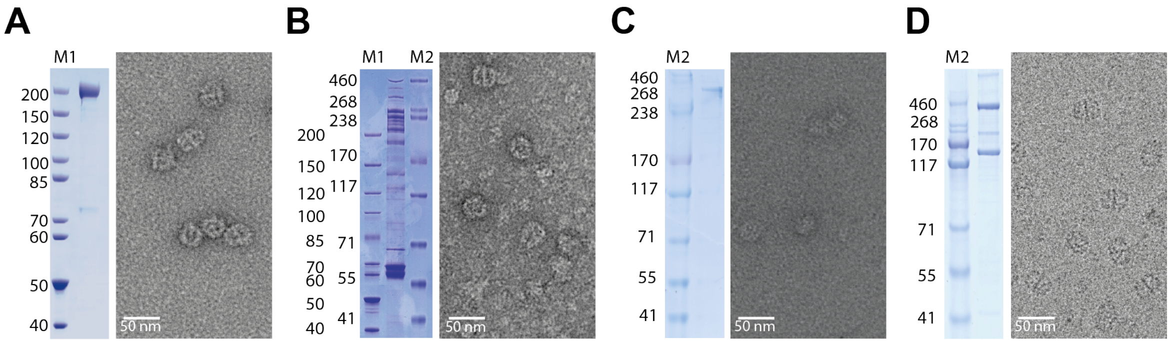

Figure S6. Electron microscopic characterization of recombinantly expressed fungal and bacterial FAS in *E. coli*. SDS-PAGE gels and negative stain micrographs are shown for recombinantly expressed *S. cerevisiae* FAS (**A**), a β/α fusion construct of *S. cerevisiae* FAS mimicking *Ustilaginomycetes*-type FAS (**B**) and *Mycobacterium tuberculosis* FAS [11] (**C**). For *R. toruloides* FAS [10] (**D**), cryo-EM data is shown. Normal weight (M1) as well as high molecular weight markers (M2) were used. SDS-PAGE in (**B**) indicates proteolytic degradation of the single-gene encoded construct during recombinant expression and protein purification.

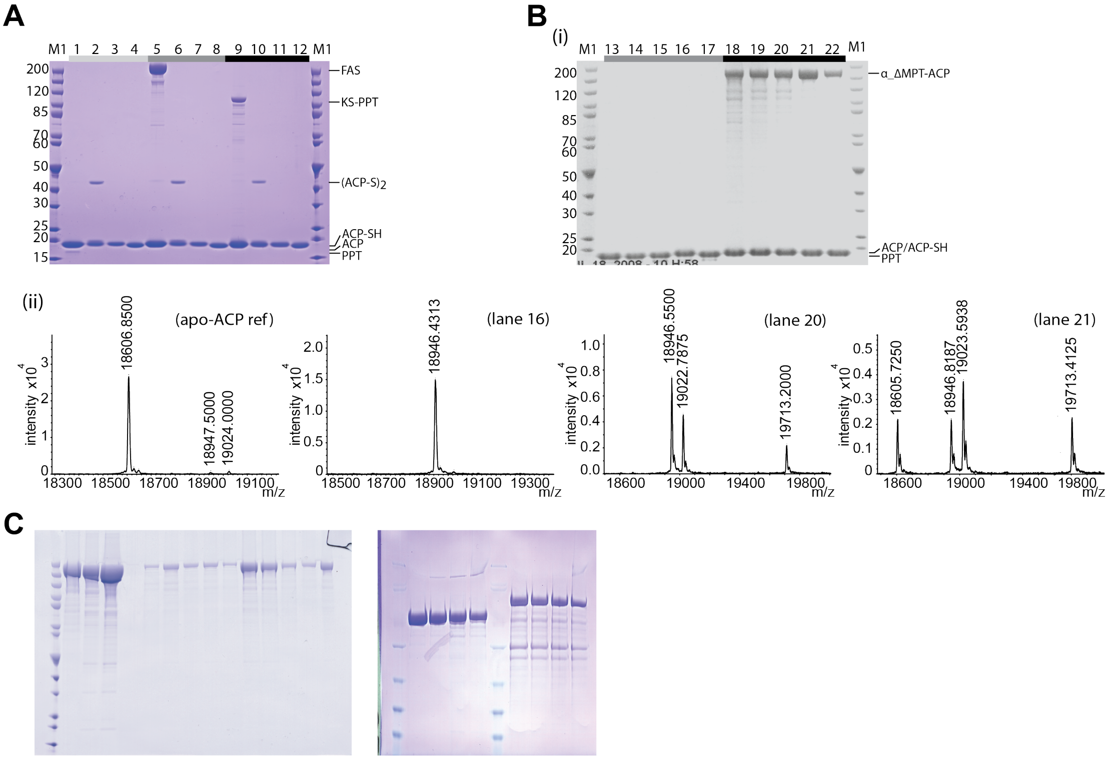

Figure S7. Phosphopantetheinylation assay with various PPT carrying constructs. A 4-12% Bis/Tris SDS-PAGE gel (NuPage, Invitrogen) showing the turn-over of apo-ACP into holo-ACP (ACP-SH) and mass spectrometric analysis of ACP modification. (**i**) Phosphopantetheinylation activity of separate PPT and $\alpha_6\beta_6$ *S. cerevisiae* FAS (lanes 1 to 8) have been reported before [2]. Lanes 9 to 12, demonstrating phosphopantetheinylation activity of the construct KS-PPT (as shown in **Figure 4B**). Lanes are colored as follows; separate PPT domain (light grey), the $\alpha_6\beta_6$ *S. cerevisiae* FAS complex (grey), and the di-domain KS-PPT (black). Lane M, marker; 1,5 & 9, reaction solution; 2, 6 & 10, ACP purified from the reaction solution and loaded on gel under non-reducing conditions stabilizing holo-ACP that dimerizes *via* a disulfide linkage (ACP-S)$_2$; 3, 7 & 11, same as 2, 6 & 10,but loaded on gel under reducing conditions, which cleaves disulfide bonds; 4, 8 & 12, ACP apoprotein reference. The turnover of ACP was monitored via mass spectrometric analysis (ESI-MS) [2]. **B** Phosphopantetheinylation assay with the separate PPT domain (lanes 13 to 17) and the α_ΔMPT-ACP construct (18 to 22). Protein that has been refolded by dialysis (lanes 18 and 19) and by size-exclusion chromatography (lanes 20 and 21). The reaction solutions are loaded under reducing conditions on the SDS-PAGE gel so that the holo-ACP, which might have dimerized *via* a disulfide linkage during phosphopantetheinylation (ACP-S)$_2$, are not stabilized. (**ii**) Mass spectrometric analysis (ESI-MSI) of reaction solutions shown in SDS-PAGE lanes 16, 20 and 21 (see (**i**)), as well as apo-ACP reference. **C** Full SDS-PAGE gels shown in cropped version in **Figure 4C**.

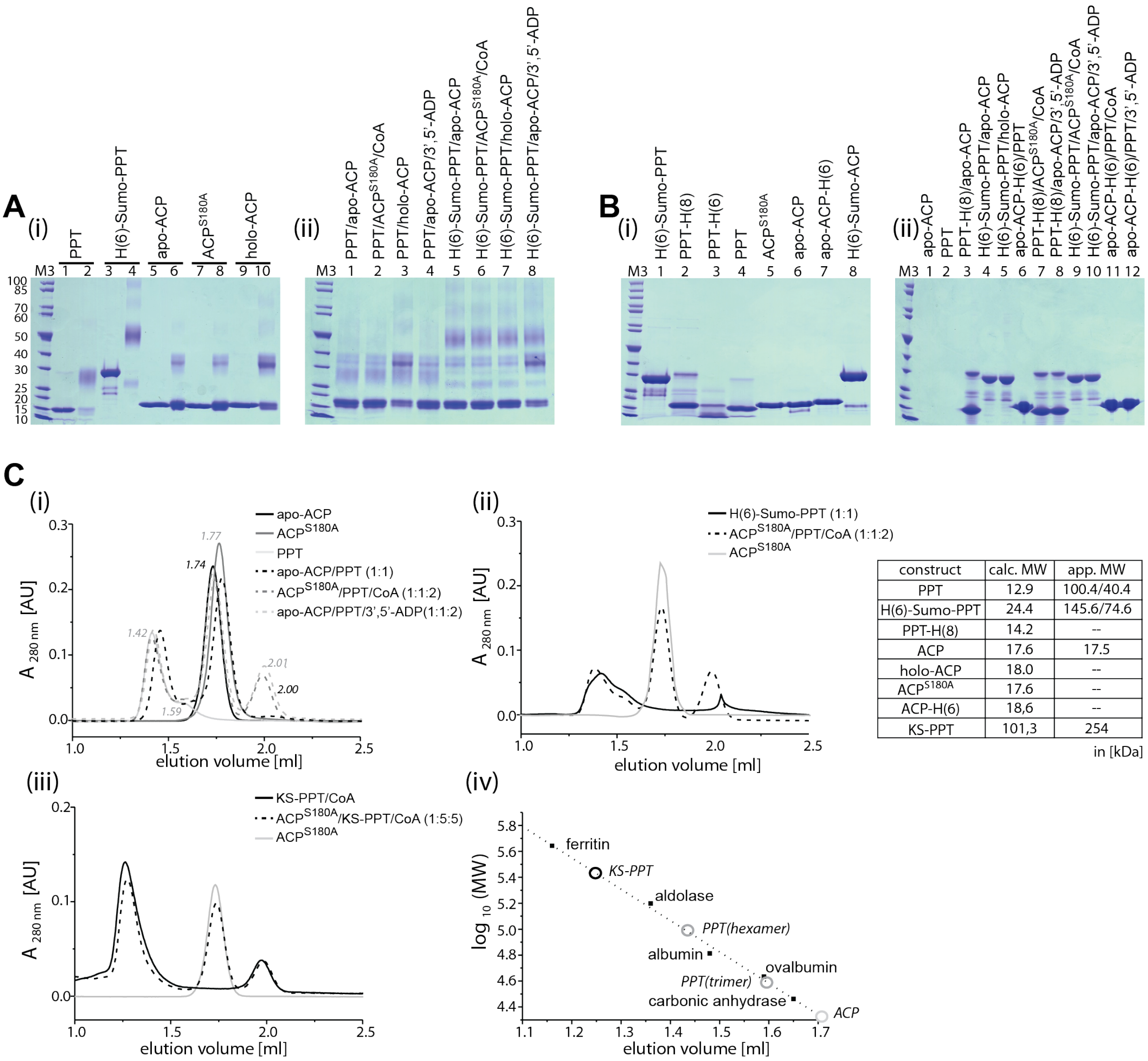

construct	calc. MW	app. MW
PPT	12.9	100.4/40.4
H(6)-Sumo-PPT	24.4	145.6/74.6
PPT-H(8)	14.2	--
ACP	17.6	17.5
holo-ACP	18.0	--
ACP^{S180A}	17.6	--
ACP-H(6)	18,6	--
KS-PPT	101,3	254

in [kDa]

Figure S8. Studies on the interaction of ACP and PPT. A Analysis of ACP-PPT interaction via glutaraldehyde-induced crosslinking. 4%-12% Bis/Tris SDS-PAGE gel (NuPAGE, Invitrogen) showing crosslinks of individual proteins and between ACP and PPT. A table of molecular weight is attached. (**i**) Control reactions with the individual proteins. Crosslinked proteins (even-numbered lanes) are shown next to untreated proteins for reference (odd-numbered lanes). Apo-ACP refers to non-phosphopantetheinylated protein, holo-ACP to phosphopantetheinylated protein, received by treatment with PPT and CoA as a substrate. The Sumo-domain should mimic N-terminal elongation of PPT as occurring as covalent part of the α-subunit. The ternary complexes (ACP and PPT constructs with ligands) were analyzed for mimicking putative states on a reaction coordinate. (**ii**) Crosslinks between ACP and PPT. Lane 1 (PPT/apo-ACP, molar ratio 1:1); 2 (PPT/ACP^{S180A}/CoA, 1:1:2), 3 (PPT/holo-ACP, 1:1); 4 (PPT/apo-ACP/3',5'-ADP, 1:1:2); 5 (H(6)-Sumo-PPT/apo-ACP, 1:1), 6 (H(6)-Sumo-PPT/ACP^{S180A}/CoA, 1:1:2); 7 (H(6)-Sumo-PPT/holo-ACP, 1:1); 8 (H(6)-Sumo-PPT/apo-ACP/3',5'ADP, 1:1:2). **B** Analysis of ACP-PPT interaction via Ni-NTA pull-downs analyzed by SDS-PAGE (4%-12% Bis/Tris SDS-PAGE gel (NuPAGE, Invitrogen)). (**i**) Proteins used for

pull-down assay; shown for reference. PPT-H(6) has not been used in this study. (**ii**) Lane 1 (apo-ACP); 2 (PPT), 3 (PPT-H(8)/ACP, molar ratio 1:1); 4 (H(6)-Sumo-PPT/apo-ACP, 1:1); 5 (H(6)-Sumo-PPT/holo-ACP, 1:1), 6 (apo-ACP_H(6)/PPT, 1:1); 7 (PPT_H(8)/ACP^{S180A}/CoA, 1:1:2); 8 (PPT_H(8)/apo-ACP/3',5'-ADP, 1:1:2), 9 (H(6)-Sumo-PPT/ACP^{S180}A/CoA, 1:1:2); 10 (H(6)-Sumo-PPT/apo-ACP/3',5'-ADP, 1:1:2), 11 (apo-ACP-H(6)/PPT/CoA, 1:1:2); 12 (apo-ACP-H(6)/PPT/3',5'-ADP, 1:1:2)). **C** Size-exclusion chromatography of individual proteins and ACP/PPT equimolar solutions on a Superdex 200 3.2/30 (GE Healthcare) column. (**i**) Superposition of chromatographic profiles of apo-ACP (black), ACP-S180A (grey) and PPT (light grey), as reference proteins (solid lines), and solutions of apo-ACP/PPT (molar ratio 1:1, black), ACP^{S180A}/PPT/CoA (1:1:2, grey), and apo-ACP/PPT/3',5'ADP (1:1:2, light grey) in dashed lines. (**ii**) SEC was also performed with sumoylated PPT, and interaction study of solution ACP^{S180A}/Sumo-PPT/CoA is shown as example (1:1:2; dashed line). Superposition of chromatographic profiles of the individual proteins Sumo-PPT (black) and ACP^{S180A} (grey) is given for reference. (**iii**) As an example for interaction studies with the KS-PPT didomain construct, superposition is shown of the chromatographic profile of solution ACP^{S180A}/KS-PPT/CoA (~1:5:5, dashed line) with profiles of the individual proteins KS-PPT with CoA (black) and ACP^{S180A} (grey). (**iv**) Linear regression of calibration with reference proteins for estimating the oligomeric state of proteins. Elution volumes of KS-PPT (black), PPT (grey) and apo-ACP (light grey) are indicated by circles. Apparent molecular weights for selected proteins, as received by SEC, are given in the attached table. Accordingly, PPT shows mixed trimeric/hexameric properties as separate of Sumo-fusion construct, KS-PPT is dimeric with an increased apparent molecular weight putatively resulting from an elongated shape and/or flexibly attached PPT [9], and ACP is monomeric.

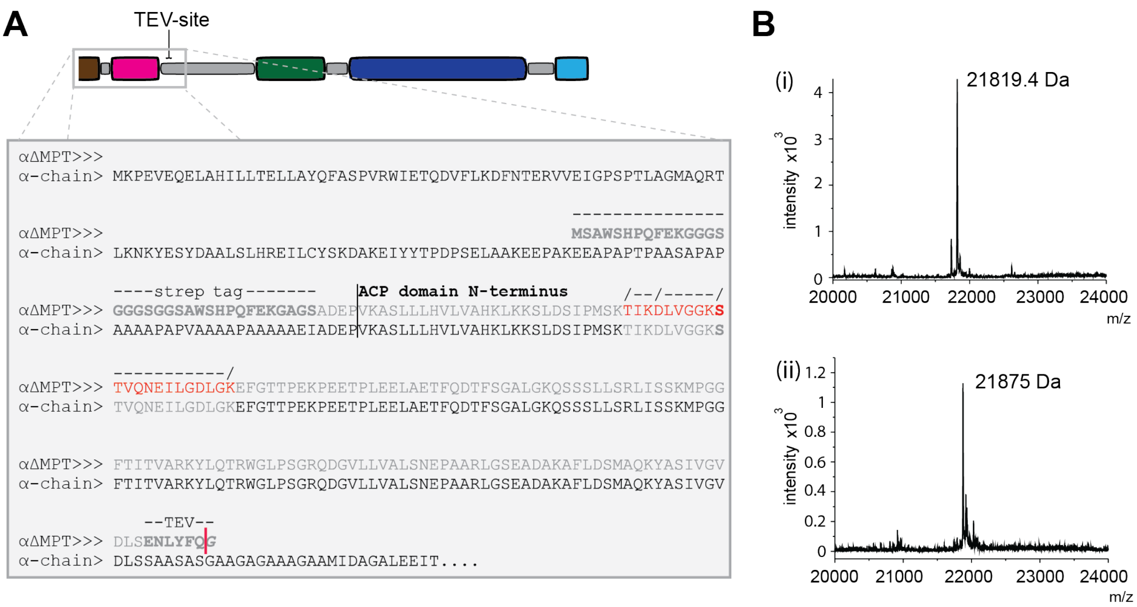

Figure S9. Mass spectrometric analysis of auto-phosphopantetheinylation of the α-subunit. A Alignment of the N-termini of the α-subunit (black) and the construct α_ΔMPT_tev. The TEV cleavage site is inserted within the flexible linker region C-terminal to the ACP domain (the preparation of α_ΔMPT_tev is described in the Material and Methods section). The peptide colored in red has been identified after tryptic in-gel-digestion of SDS-PAGE. Miscleavages are indicated by slashes. Active serine S180 is shown in bold. The peptide containing S180 (STVQNEILGDLGK) was identified, and determined to have a +397 mass shift consistent with phosphopantetheine (+340) covalently attached to Ser and carbamidomethyl (+57) from iodacetamide mediated modification of the terminal thiol group (holo-peptide 1769.8271 Da observed, 1373.53 + 57.0513 + 340.3330 (1770.91) calculated). The apo-peptide with a calculated mass of 1373.73 Da was not detected. Data for the single- and double-miscleaved peptide containing S180 (single: DLVGGKSTVQNEILGDLGK, holo-peptide 2339.1444 Da observed, 2340.56 calculated; double: TIKDLVGGKSTVQNEILGDLGK, holo-peptide 2681.3711 Da observed, 2683.00 calculated) indicates also partially digested peptides. The apo-peptides were not detected (1943.04 and 2285.27 Da). **B** (**i**) ESI-MS analysis of cleaved ACP-containing fragments of α_ΔMPT_tev. Enrichment of α_ΔMPT_tev by Ni-chelating chromatography, incubation with TEV protease and selection of the N-terminal fragment by with Strep-Tactin affinity chromatography [3] allowed detecting the phosphopantetheinylated N-terminus of construct α_ΔMPT_tev (N-terminal fragment with holo-ACP 21819.4 (21819.3 Da, calculated); N-terminal fragment with apo-ACP (21479 Da calculated) is not visible). (**ii**) Treatment of the strep-tactin elution fractions with iodacetamide shifts the protein mass by 57 Da corresponding to carbamidomethylation of the phosphopantetheine at S180 (21876.3 Da calculated). Cysteins, as alternative targets of iodacetamide modification, are not present in the cleaved fragment. The preparation of α_ΔMPT_tev and its proteolytic cleavage is described in the Material and Methods section.

SI Tables

Table S1. Proteins produced in *E. coli*. Plasmids used in this study.

Proteins produced in E. coli and respective plamids			
**Protein name**	**Plasmid name**	**Backbone**	**Gene(construct borders)**
FAS^{rc}	pFAS1_FAS2	pETDuet-1 (Novagen)	*FAS1* and *FAS2*
FAS_β-α	pFAS12	pETDuet-1	*FAS1-FAS2*
α_ΔMPT	pFAS2_ΔMPT	pET22b(+) (Novagen)	*FAS2*(141-1887)
α_ΔMPT-tev	pFAS2_ΔMPT_tev	pET22b(+)	*FAS2*(141-1887)
α_ΔMPT-ACP	pFAS2_ΔMPT-ACP	pET22b(+)	*FAS2*(328-1887)
GST-KS-PPT	pFAS2_KS-PPT	pGEX (GE Healthcare)	*FAS2*(982-1887)
H(6)-Sumo-ACP/ ACP (after cut)	pSumo-ACP	pET28M-Sumo1*	
ACP-H(6)	pACP	pET22b(+)	*FAS2*(140-302)
(6)-Sumo-ACP^{S180A}/ ACP^{S180A} (after cut)	pSumo-ACP^{S180A}	pET28M-Sumo1	*FAS2*(140-302)
ACP^{S180A}-H(6)	pACP^{S180A}	pET22b(+)	*FAS2*(140-302)
PPT-H(8)	pPPT	pET22b(+)	*FAS2*(1766-1887)
H(6)-Sumo-PPT/ PPT (after cut)	pSumo-PPT	pET28M-Sumo1	*FAS2*(1766-1887)
S. cerevisiae plasmids			
	**Plasmid name**	**Backbone**	**Gene(construct borders)**
	pMF001	pRS416	*FAS1 and FAS2*
	pMF012	pRS315	*FAS1*
	pMF014	pRS315	*FAS1-Strep-I*
	pMF021	pRS315	*fas1 (1-2021)*
	pMF013	pRS315	*FAS2*
	pMF011	pRS313	*FAS2*
	pMF029	pRS313	*fas2_K2S*
	pMF030	pRS313	*fas2_E8R*
	pMF031	pRS313	*fas2_K2S/E8R*
	pMF050	pRS313	*fas2_E6V*
	pMF051	pRS313	*fas2_H11A*
	pMF035	pRS313	*fas2_E6V/H11A*
	pMF032	pRS313	*fas2_K2S/E6V*
	pMF033	pRS313	*fas2_K2S/H11A*
	pMF034	pRS313	*fas2_E6V/E8R*
	pMF015	pRS313	*fas2_S180A*
	pMF022	pRS313	*fas2(12-1887)*
	pMF016	pRS313	*fas1-fas2*
	pMF026	pRS315	*fas1-fas2 (3-20)*
	pMF027	pRS313	*fas1-fas2 (3-94)*
	pMF017	pRS313	*fas1-fas2 (3-427)*
	pMF036	pRS313	*fas1-fas2 (3-427)_E2058R*
	pMF018	pRS315	*fas2 (428-1887)*
	pMF023	pRS315	*fas2 (495-1887)*
	pMF028	pRS315	*fas2 (404-1887)*
	pMF019	pRS315	*fas1 (1-1201)*
	pMF020	pRS313	*fas1 (1202-2047)-fas2*
	pMF024	pRS313	*fas1 (1255-2047)-fas2*
	pMF025	pRS315	*fas1_ΔTM(2-137)*
	pBAR	pRS313	*fas2_ ΔDM2(541-598)*

Table S2. Growth behavior of mutated strains. Growth curves in liquid cultures supplemented with external FA given in numbers. Mean OD(600) with threefold standard deviation of 5 individual technical replicates per selected single clone. The error for the mean of biological replicates was calculated with Gaussian error propagation.

Strain	Clone	FA Concentration [mg/L]													
		50		35		20		15		10		5		0	
		OD_{600}	ΔOD_{600}	OD_{600}	ΔOD_{600}	OD_{600}	ΔOD_{600}	OD_{600}	ΔOD_{600}	OD_{600}	ΔOD_{600}	OD_{600}	ΔOD_{600}	OD_{600}	ΔOD_{600}
Sc_Δα67/68	C1	22,6	0,4	21,5	0,6	12,5	0,4	9,5	0,3	6,1	0,2	2,8	0,1	0,1	0,1
	C2	22,3	0,4	19,8	0,3	12,7	0,2	9,0	0,4	5,9	0,2	2,8	0,1	0,1	0,3
	**mean**	**22,5**	**0,5**	**20,7**	**0,7**	**12,6**	**0,5**	**9,2**	**0,5**	**6,0**	**0,3**	**2,8**	**0,1**	**0,1**	**0,3**
Sc_Δα1(2-11)	C1	27,8	0,6	20,9	0,5	13,7	0,9	10,0	0,6	7,2	0,2	3,9	0,3	0,1	0,1
	C2	25,2	0,4	22,6	2,4	12,3	0,4	9,5	0,4	6,6	0,4	3,1	0,3	0,1	0,1
	**mean**	**26,5**	**0,7**	**21,7**	**2,5**	**13,0**	**1,0**	**9,7**	**0,7**	**6,9**	**0,4**	**3,5**	**0,4**	**0,1**	**0,2**
Sc_K2S	C1	25,3	0,2	23,5	0,2	21,6	0,6	21,5	0,4	20,3	0,7	19,9	0,2	19,6	0,5
	C2	27,0	0,3	24,0	0,7	25,7	0,3	21,0	0,3	21,1	0,2	20,1	0,3	20,8	0,3
	**mean**	**26,2**	**0,4**	**23,7**	**0,7**	**23,7**	**0,6**	**21,2**	**0,5**	**20,7**	**0,7**	**20,0**	**0,4**	**20,2**	**0,5**
Sc_E8R	C1	26,9	0,5	25,5	0,3	23,7	0,4	21,1	0,4	20,7	0,6	19,9	0,7	20,8	0,3
	C2	24,4	0,3	24,8	0,6	22,6	0,3	21,7	0,9	20,4	0,1	20,6	0,2	20,2	0,3
	**mean**	**25,6**	**0,6**	**25,1**	**0,7**	**23,1**	**0,5**	**21,4**	**1,0**	**20,5**	**0,7**	**20,2**	**0,8**	**20,5**	**0,4**
Sc_K2S/E8R	C1	22,7	0,9	21,1	0,6	15,2	0,4	10,6	0,6	7,6	0,4	3,8	0,3	0,2	0,3
	C2	22,8	0,7	20,5	0,2	15,6	0,5	10,3	0,8	7,7	0,6	3,3	0,6	0,2	0,3
	**mean**	**22,8**	**1,1**	**20,8**	**0,6**	**15,4**	**0,6**	**10,4**	**1,0**	**7,6**	**0,7**	**3,5**	**0,7**	**0,2**	**0,4**
Sc_KO	C1	33,8	0,7	26,5	0,5	21,3	0,9	14,2	0,6	8,6	0,4	4,4	0,1	0,2	0,1
	C2	32,4	0,5	27,5	0,2	21,4	1,2	15,0	0,2	9,9	0,4	4,7	0,2	0,4	0,1
	**mean**	**33,1**	**0,8**	**27,0**	**0,5**	**21,4**	**1,5**	**14,6**	**0,6**	**9,3**	**0,6**	**4,5**	**0,2**	**0,3**	**0,2**
Sc_WT	C1	35,5	1,0	34,5	0,6	32,5	0,9	33,2	0,2	32,8	0,4	32,2	1,3	30,9	0,3
	C2	31,6	4,4	30,4	5,1	28,5	3,8	27,0	4,4	25,5	3,1	25,3	3,9	25,1	3,8
	C3	32,1	4,8	33,0	3,9	30,4	2,3	27,8	5,2	26,0	4,5	27,3	2,7	25,4	2,5
	**mean**	**31,8**	**6,5**	**31,7**	**6,4**	**29,5**	**4,4**	**27,4**	**6,8**	**25,7**	**5,5**	**26,3**	**4,7**	**25,2**	**4,6**

Table S2 (continued). Growth behavior of mutated strains. Growth curves in liquid cultures supplemented with external FA given in numbers. Mean OD(600) with threefold standard deviation of 5 individual technical replicates per selected single clone. The error for the mean of biological replicates was calculated with Gaussian error propagation.

Strain	Clone	FA Concentration [mg/L]													
		50		35		20		15		10		5		0	
		OD_{600}	ΔOD_{600}	OD_{600}	ΔOD_{600}	OD_{600}	ΔOD_{600}	OD_{600}	ΔOD_{600}	OD_{600}	ΔOD_{600}	OD_{600}	ΔOD_{600}	OD_{600}	ΔOD_{600}
Sc_Tre_E8R	C1	31,0	0,4	29,5	0,3	28,6	0,9	27,3	0,4	26,1	0,4	26,0	0,3	26,3	0,7
	C2	28,2	0,4	28,3	0,8	26,2	0,5	25,8	0,6	26,2	0,4	26,7	0,6	26,4	0,4
	**mean**	29,6	0,6	28,9	0,9	27,4	1,0	26,5	0,7	26,2	0,5	26,4	0,7	26,3	0,8
Sc_Tre_E8R	C1	35,6	5,6	32,3	5,1	29,4	7,6	29,4	2,7	29,1	5,3	29,6	2,8	29,9	6,7
	C2	33,3	3,8	29,1	3,2	28,1	5,9	27,8	2,9	25,4	4,8	27,0	1,5	26,9	6,0
	**mean**	34,5	6,7	30,7	6,0	28,7	9,6	28,6	4,0	27,2	7,1	28,3	3,2	28,4	8,9
Sc_ E6V/H11A	C1	23,1	0,5	23,0	1,1	21,8	0,5	20,3	0,7	19,9	0,8	20,5	0,5	18,2	0,3
	C2	24,3	0,3	23,1	0,6	21,4	0,5	21,1	0,4	19,7	0,6	20,1	0,6	19,2	0,5
	**mean**	23,7	0,6	23,0	1,2	21,6	0,7	20,7	0,8	19,8	1,0	20,3	0,8	18,7	0,6
Sc_ K2S/E6V	C1	24,2	0,9	20,9	0,5	20,5	0,7	19,5	0,7	18,5	0,6	18,8	0,3	17,0	0,2
	C2	24,0	0,5	22,2	0,4	20,5	0,7	19,6	0,8	18,4	0,3	19,2	0,3	17,1	0,3
	**mean**	24,1	1,0	21,5	0,6	20,5	1,0	19,5	1,1	18,5	0,7	19,0	0,4	17,1	0,4
Sc_ K2S/E6V	C1	24,4	0,4	24,1	0,7	22,4	0,2	22,8	0,7	22,7	0,2	21,2	0,4	22,0	0,4
	C2	25,9	0,7	24,5	0,7	23,2	0,3	22,2	0,3	21,9	0,3	20,9	0,5	21,7	0,4
	**mean**	25,1	0,7	24,3	1,0	22,8	0,3	22,5	0,7	22,3	0,4	21,1	0,7	21,9	0,6
Sc_ E8R/E6V	C1	24,6	0,5	23,0	0,4	21,3	0,3	21,0	0,3	20,2	0,3	20,4	0,5	21,3	0,3
	C2	24,3	0,7	24,0	0,7	22,3	0,9	20,7	0,7	19,9	0,3	20,0	0,3	20,5	0,1
	**mean**	24,5	0,8	23,5	0,8	21,8	1,0	20,8	0,8	20,1	0,5	20,2	0,6	20,9	0,3

Table S2 (continued). Growth behavior of mutated strains. Growth curves in liquid cultures supplemented with external FA given in numbers. Mean OD(600) with threefold standard deviation of 5 individual technical replicates per selected single clone. The error for the mean of biological replicates was calculated with Gaussian error propagation.

**Strain**	**Clone**	**FA Concentration [mg/L]**													
		**50**		**35**		**20**		**15**		**10**		**5**		**0**	
		OD_{600}	ΔOD_{600}	OD_{600}	ΔOD_{600}	OD_{600}	ΔOD_{600}	OD_{600}	ΔOD_{600}	OD_{600}	ΔOD_{600}	OD_{600}	ΔOD_{600}	OD_{600}	ΔOD_{600}
Sc_fas1-fas2	C1	23,6	0,8	23,9	0,3	23,8	0,4	23,4	0,3	22,9	0,5	21,2	0,2	21,4	0,8
	C2	23,7	0,3	26,7	0,6	22,7	0,3	23,4	0,8	24,0	3,3	21,6	0,6	21,4	0,6
	**mean**	23,6	0,9	25,3	0,7	23,3	0,5	23,4	0,8	23,4	3,3	21,4	0,6	21,4	1,0
Sc_Tre	C1	26,3	0,6	25,5	0,6	21,7	0,6	21,6	0,3	21,1	0,2	19,6	0,6	18,6	1,4
	C2	25,1	0,4	26,4	0,6	23,4	0,9	22,1	0,4	20,5	0,5	20,1	0,6	19,3	0,2
	**mean**	25,7	0,8	25,9	0,9	22,5	1,1	21,9	0,5	20,8	0,5	19,9	0,8	18,9	1,4
Sc_Rho	C1	25,1	0,5	25,0	0,4	22,5	0,4	22,1	0,8	20,1	0,6	18,8	0,6	19,0	0,7
	C2	25,8	0,3	25,0	0,3	23,5	0,6	21,8	0,4	21,0	0,4	19,3	0,3	21,0	0,4
	**mean**	25,4	0,6	25,0	0,5	23,0	0,7	21,9	0,9	20,5	0,7	19,1	0,7	20,0	0,8
Sc_fas1-full-MPT	C1	24,6	0,5	23,0	0,4	14,6	0,5	9,5	0,2	6,9	0,3	3,4	0,1	0,0	0,1
	C2	24,6	0,8	22,6	0,6	13,8	0,4	10,5	0,3	6,8	0,4	3,0	0,3	0,0	0,1
	**mean**	24,6	1,0	22,8	0,7	14,2	0,6	10,0	0,4	6,8	0,5	3,2	0,3	0,0	0,1
Sc_ fas2Tr-α9	C1	23,9	0,3	22,8	0,5	16,2	0,2	12,4	0,8	10,4	0,6	6,3	0,4	1,5	0,5
	C2	24,1	0,4	23,2	0,5	16,2	0,2	12,0	0,4	10,5	0,2	6,4	0,2	1,1	0,2
	**mean**	24,0	0,5	23,0	0,7	16,2	0,3	12,2	0,9	10,5	0,6	6,3	0,5	1,3	0,5
Sc_ fas1-α1	C1	22,8	0,5	22,9	0,9	17,8	0,3	16,4	0,3	14,0	0,3	13,4	0,2	12,4	0,3
	C2	26,2	0,4	21,4	0,4	17,7	0,9	16,9	1,1	14,4	0,1	13,9	0,2	11,7	0,2
	**mean**	24,5	0,6	22,1	1,0	17,8	0,9	16,7	1,1	14,2	0,3	13,7	0,3	12,1	0,3
Sc_Tre_ Δα10-12	C1	27,7	0,3	24,4	0,3	19,5	0,7	11,6	0,5	6,6	0,4	2,4	0,3	0,3	0,2
	C2	25,6	0,4	26,8	0,2	17,8	0,3	10,1	0,1	6,0	0,3	2,5	0,3	0,4	0,2
	**mean**	26,6	0,4	25,6	0,4	18,6	0,8	10,8	0,5	6,3	0,5	2,4	0,4	0,3	0,3
Sc_Rho_ Δ(1-53)	C1	23,6	1,0	24,3	0,6	18,6	0,5	10,3	0,5	7,0	0,5	2,6	0,4	0,4	0,3
	C2	25,0	0,5	25,0	0,3	17,6	0,2	9,3	0,4	6,7	0,4	2,7	0,2	0,2	0,3
	**mean**	24,3	1,1	24,7	0,7	18,1	0,5	9,8	0,6	6,8	0,6	2,7	0,4	0,3	0,4
Sc_ Asc/Tre	C1	24,1	0,3	22,7	1,0	16,5	0,4	13,5	0,4	13,5	0,4	12,8	0,6	11,4	0,3
	C2	22,9	0,4	21,7	0,5	16,3	0,8	13,4	0,6	14,3	0,7	11,6	0,6	11,2	0,4
	**mean**	23,5	0,5	22,2	1,1	16,4	0,9	13,4	0,8	13,9	0,9	12,2	0,9	11,3	0,5

SI REFERENCES

1 Chirala, S. S. Coordinated regulation and inositol-mediated and fatty acid-mediated repression of fatty acid synthase genes in Saccharomyces cerevisiae. *Proc. Natl. Acad. Sci. U. S. A.* **89**, 10232-10236, doi:10.1073/pnas.89.21.10232 (1992).

2 Johansson, P. *et al.* Multimeric options for the auto-activation of the Saccharomyces cerevisiae FAS type I megasynthase. *Structure* **17**, 1063-1074 (2009).

3 Schmidt, T. & Skerra, A. The Strep-tag system for one-step purification and high-affinity detection or capturing of proteins. *Nat Protoc.* **2**, 1528-1535 (2007).

4 Gietz, R. D. & Schiestl, R. H. High-efficiency yeast transformation using the LiAc/SS carrier DNA/PEG method. *Nat Protoc* **2**, 31-34, doi:10.1038/nprot.2007.13 (2007).

5 Johansson, P. *et al.* Inhibition of the fungal fatty acid synthase type I multienzyme complex. *Proc Natl Acad Sci U S A.* **105**, 12803-12808 (2008).

6 Egner, R. *et al.* Tracing intracellular proteolytic pathways. Proteolysis of fatty acid synthase and other cytoplasmic proteins in the yeast Saccharomyces cerevisiae. *J Biol Chem* **268**, 27269-27276 (1993).

7 Leibundgut, M., Jenni, S., Frick, C. & Ban, N. Structural basis for substrate delivery by acyl carrier protein in the yeast fatty acid synthase. *Science* **316**, 288-290 (2007).

8 Oefner, C., Schulz, H., D'Arcy, A. & Dale, G. E. Mapping the active site of Escherichia coli malonyl-CoA-acyl carrier protein transacylase (FabD) by protein crystallography. *Acta Crystallogr D Biol Crystallogr* **62**, 613-618, doi:10.1107/s0907444906009474 (2006).

9 Jenni, S. *et al.* Structure of fungal fatty acid synthase and implications for iterative substrate shuttling. *Science* **316**, 254-261 (2007).

10 Fischer, M. *et al.* Cryo-EM structure of fatty acid synthase (FAS) from Rhodosporidium toruloides provides insights into the evolutionary development of fungal FAS. *Protein Sci* **24**, 987-995, doi:10.1002/pro.2678 (2015).

11 Ciccarelli, L. *et al.* Structure and conformational variability of the Mycobacterium tuberculosis fatty acid synthase multienzyme complex. *Structure* **21**, 1251-1257 (2013).
